# Identification and Molecular Characterization of the Switchgrass AP2/ERF Transcription Factor Superfamily, and Overexpression of *PvERF001* for Improvement of Biomass Characteristics for Biofuel

**DOI:** 10.3389/fbioe.2015.00101

**Published:** 2015-07-20

**Authors:** Wegi A. Wuddineh, Mitra Mazarei, Geoffrey B. Turner, Robert W. Sykes, Stephen R. Decker, Mark F. Davis, C. Neal Stewart

**Affiliations:** ^1^Department of Plant Sciences, University of Tennessee, Knoxville, TN, USA; ^2^Bioenergy Science Center, Oak Ridge National Laboratory, Oak Ridge, TN, USA; ^3^National Renewable Energy Laboratory, Golden, CO, USA

**Keywords:** AP2, ethylene response factors, stress response, transcription factors, biofuel, PvERF001, overexpression, sugar release

## Abstract

The APETALA2/ethylene response factor (AP2/ERF) superfamily of transcription factors (TFs) plays essential roles in the regulation of various growth and developmental programs including stress responses. Members of these TFs in other plant species have been implicated to play a role in the regulation of cell wall biosynthesis. Here, we identified a total of 207 AP2/ERF TF genes in the switchgrass genome and grouped into four gene families comprised of 25 AP2-, 121 ERF-, 55 DREB (dehydration responsive element binding)-, and 5 RAV (related to API3/VP) genes, as well as a singleton gene not fitting any of the above families. The ERF and DREB subfamilies comprised seven and four distinct groups, respectively. Analysis of exon/intron structures of switchgrass AP2/ERF genes showed high diversity in the distribution of introns in AP2 genes versus a single or no intron in most genes in the ERF and RAV families. The majority of the subfamilies or groups within it were characterized by the presence of one or more specific conserved protein motifs. *In silico* functional analysis revealed that many genes in these families might be associated with the regulation of responses to environmental stimuli via transcriptional regulation of the response genes. Moreover, these genes had diverse endogenous expression patterns in switchgrass during seed germination, vegetative growth, flower development, and seed formation. Interestingly, several members of the ERF and DREB families were found to be highly expressed in plant tissues where active lignification occurs. These results provide vital resources to select candidate genes to potentially impart tolerance to environmental stress as well as reduced recalcitrance. Overexpression of one of the ERF genes (*PvERF001*) in switchgrass was associated with increased biomass yield and sugar release efficiency in transgenic lines, exemplifying the potential of these TFs in the development of lignocellulosic feedstocks with improved biomass characteristics for biofuels.

## Introduction

Switchgrass (*Panicum virgatum*) is an outcrossing perennial C4 grass known for its vigorous growth and wide adaptability and, hence, is being developed as a candidate lignocellulosic biofuel feedstock (Yuan et al., 2008). The feasibility of commercial production of liquid transportation biofuel from switchgrass biomass is hampered by biomass recalcitrance (the resistance of cell wall to enzymatic breakdown into simple sugars). Lignin is considered to be a primary contributor to biomass recalcitrance as it hinders the accessibility of cell wall carbohydrates to hydrolytic enzymes. Substantial progress has been made in engineering the switchgrass lignin biosynthesis pathway to reduce lignin content and/or modify its composition (Fu et al., 2011a,b; Shen et al., 2012, 2013a,b; Baxter et al., 2014, 2015). The downregulation of individual genes in the lignin biosynthesis pathway has been effective to reduce lignin, but can result in the production of metabolites that can impede downstream fermentation processes (Tschaplinski et al., 2012). Alternatively, overexpression of transcription factors (TFs), such as switchgrass *MYB4*, has been shown to circumvent this inhibitory effect while leading to significantly reduced biomass recalcitrance and improved ethanol production (Shen et al., 2012, 2013b; Baxter et al., 2015).

The master regulators of gene cluster TFs with altered expression could, in turn, endow such traits as increased biomass yield, tiller number, improved germination/plant establishment, or root growth as well as tolerance to environmental stresses (Xu et al., 2011; Licausi et al., 2013; Ambavaram et al., 2014). Therefore, identification of TFs with such putative roles would provide a dynamic approach to developing better biofuel feedstocks that could thrive under adverse environmental conditions. The availability of switchgrass ESTs (Zhang et al., 2013) and draft genome sequences produced by Joint Genome Institute (JGI), Department of Energy, USA, provides a vital resource for the discovery of relevant target genes that could be utilized in the genetic improvement of perennial grasses, which could be used as dedicated bioenergy feedstocks. However, compared to dicots such as *Arabidopsis*, relatively little is known about the key regulatory mechanisms in monocots that control lignification and cell wall formation; this is especially true of switchgrass. Likewise, we also have depauperate knowledge about stress responses and defense against pests in these species.

APETALA2/ethylene responsive factor (AP2/ERF) is a large group of regulatory protein families in plants that are characterized by the presence of one or two conserved AP2 DNA binding domains. AP2/ERF TFs are involved in the transcriptional regulation of various growth and developmental processes and responses to environmental stressors. The AP2 domain is a stretch of 60–70 conserved amino acid sequences that is essential for the activity of AP2/ERF TFs (Jofuku et al., 2005). It has been demonstrated that the AP2 domain binds the *cis*-acting elements including the GCC box motif (Ohme-Takagi and Shinshi, 1995), the dehydration responsive element (DRE)/C-repeat element (CRT) (Sun et al., 2008), and/or TTG motif (Wang et al., 2015) present in the promoter regions of target genes thereby regulating their expression. The AP2/ERF superfamily can be divided into three major families, namely ERF, AP2, and RAV (related to API3/VP) (Licausi et al., 2013). The ERF family is further subdivided into two subfamilies, ERF and dehydration responsive element binding proteins (DREB) based on similarities in amino acid residues in the AP2 domain. The DREB subfamily in *Arabidopsis* and rice has been further classified into 4 distinct groups while ERF subfamily was clustered into 8 groups in *Arabidopsis* and 11 groups in rice based on analysis of gene structure and conserved motifs (Nakano et al., 2006). The AP2 family comprises two groups of proteins differing in the number of AP2 domain in their amino acid sequences. The majority of proteins in this group are characterized by the presence of two AP2 domains, but a few members of this group have only a single AP2 domain that is more similar to the AP2 domains in the double domain groups. RAV proteins, on the other hand, are a small family TFs characterized by the presence of B3 DNA binding domain besides a single AP2 domain. Genome-wide analysis of AP2/ERF TFs has been extensively studied in many dicots including *Arabidopsis* (Nakano et al., 2006), *Populus* (Zhuang et al., 2008; Vahala et al., 2013), Chinese cabbage (Liu et al., 2013), grapevine (Licausi et al., 2010), peach (Zhang et al., 2012), and castor bean (Xu et al., 2013). However, with the exception of rice (Nakano et al., 2006; Rashid et al., 2012), and foxtail millet (Lata et al., 2014), little information is available on the AP2/ERF TF families in monocots such as switchgrass.

Numerous genes coding for AP2/ERF superfamily TFs have been identified and functionally characterized in various plant species (Xu et al., 2011; Licausi et al., 2013). The DREB subfamily proteins have been extensively studied with regard to tolerance to abiotic stress such as freezing (Jaglo-Ottosen et al., 1998; Ito et al., 2006; Fang et al., 2015), drought (Hong and Kim, 2005; Oh et al., 2009; Fang et al., 2015), heat (Qin et al., 2007), and salinity (Hong and Kim, 2005; Bouaziz et al., 2013). Moreover, it has been reported that DREB genes play roles in the regulation of ABA-mediated gene expression in response to osmotic stress during germination and early vegetative growth stage (Fujita et al., 2011). ERF TFs, on the other hand, have been shown to participate in the regulation of defense responses against various biotic stresses (Guo et al., 2004; Dong et al., 2015) and/or tolerance to environmental stressors, such as drought (Aharoni et al., 2004; Zhang et al., 2010b), osmotic stress (Zhang et al., 2010a), salinity (Guo et al., 2004), hypoxia (Hattori et al., 2009), and freezing (Zhang and Huang, 2010). Moreover, AP2/ERF TFs in aspen (PtaERF1) and *Arabidopsis* (AtERF004 and AtERF038) have been suggested to be associated with the regulation of cell wall biosynthesis in some tissues (Van Raemdonck et al., 2005; Lasserre et al., 2008; Ambavaram et al., 2011). The functions of AP2 family TFs, on the other hand, have been associated with plant organ-specific regulation of growth and developmental programs (Elliott et al., 1996; Jofuku et al., 2005; Horstman et al., 2014). Genes in the RAV TF family have been shown to play a role in the regulation of gene expression in response to phytohormones such as ethylene and brassinosteroid as well as in response to biotic and abiotic stresses (Mittal et al., 2014). Therefore, AP2/ERF TF superfamily may hold tremendous potential for the improvement of bioenergy feedstocks, such as switchgrass, that is intended to be grown on marginal lands that could impose undue environmental stress.

In this study, we report the identification of 207 AP2/ERF TF genes in the switchgrass genome. Cluster analysis of the identified proteins, distribution of conserved motifs, analysis of their gene structure, and expression profiling were presented. We highlight the potential application of these data to identify putative target genes that might be exploited to improve bioenergy feedstocks. To that end, we cloned one of the ERF subfamily genes, which was subsequently overexpressed in switchgrass to improve biomass productivity and sugar release efficiency.

## Materials and Methods

### Identification of AP2/ERF gene families in switchgrass genome

We used representative genes from appropriate rice gene families as the basis to search for orthologs in switchgrass. The amino acid sequences of AP2 domain-containing rice genes represented three families: AP2 (Os02g40070), ERF (Os06g40150), and RAV (Os01g04800). These proteins were used to query the derived amino acid sequences of all switchgrass AP2/ERF TFs using tblastn against the switchgrass EST database (Zhang et al., 2013) or blastp against the *P. virgatum* draft genome (Phytozome v1.1 DOE-JGI)^1^. The sequences were retrieved and evaluated for the presence of AP2 domains by searching against the conserved domain database (CDD) at NCBI. The AP2-containing switchgrass sequences were further evaluated for any redundant and missing sequences by blastp searches using the previously identified homologous counterparts of the foxtail millet (Lata et al., 2014) and rice (Nakano et al., 2006; Rashid et al., 2012). The presence of multiple gene copies from the tetraploid switchgrass genome was addressed by the identification of only a single gene copy with the highest similarity to the corresponding homologs in foxtail millet or rice. Genes with additional domains besides the AP2 domain with no corresponding homologs in foxtail millet, rice, and *Arabidopsis* AP2/ERF TFs were excluded from our subsequent analysis.

### Cluster and protein sequence analysis of AP2/ERF TFs

The amino acid sequences of the AP2/ERF TFs were imported into the MEGA6 program and multiple sequence alignment analysis was conducted using MUSCLE with default parameters (Edgar, 2004). Construction of cluster trees was performed using the neighbor-joining (NJ) method by the MEGA6 program using a bootstrap value of 1000, Poisson correction and pairwise deletion (Tamura et al., 2013). Conserved motifs in switchgrass AP2/ERF TFs were identified with the online tool, MEME version 4.10.0^2^ using the following parameters: optimum width, 6–200 amino acids; with any number of repetitions and maximum number of motifs set at 25 (Bailey and Elkan, 1994).

### Analysis of gene structure and gene ontology annotation

The genomic and coding DNA sequences of the identified AP2/ERF TFs were retrieved from the Phytozome (*P. virgatum* v1.1 DOE-JGI). The exon–intron organizations in these genes were visualized by the gene structure display server^3^ (Guo et al., 2007). To evaluate the gene ontology (GO) annotation of the identified AP2/ERF TFs, their amino acid sequences were imported into the Blast2GO suite (Conesa and Gotz, 2008). Blastp search was performed against rice protein sequences at NCBI. The resulting hits were mapped to obtain the GO terms, which were annotated to assign functional terms to the query sequences. Plant GOslim was used to filter the annotation to plant-related terms. The protein subcellular localization prediction tool WOLF PSORT^4^ was used to complement the results of the cellular localization predicted by blast2GO.

### Analysis of transcript data from the switchgrass gene expression atlas

The transcript data for the AP2/ERF superfamily TFs were extracted from the publicly available switchgrass gene expression atlas (PviGEA)^5^ (Zhang et al., 2013), which was obtained by Affymetrix microarray analysis. The probe set IDs of 108 matching genes representing the switchgrass unitranscripts (PviUT) were identified by tblastn query search using the amino acid sequences of the AP2/ERF TFs. The transcript data for each tissues and stage of development were retrieved using the probe set IDs. The expression values of the genes were log2 transformed and a heatmap was created using an online graphing tool, Plotly^6^. Tissues used for the extraction of RNA to determine the level of expression included the following: whole seeds for seed germination at 24, 48, 72 and 96 h intervals post-imbibition, whole shoots and roots at vegetative stages, V1–V5, pooled leaf sheath (LSH), leaf blade (LB) and nodes, whole crown, the bottom, middle, and top portions of the fourth internode, vascular bundle tissues, and middle portion of the third internode all at E4 (stem elongation stage 4) developmental stage. For analysis of the expression level during reproductive developmental stages, inflorescence tissues and whole seeds along with floral tissues such as lemma and palea were used.

### Vector construction and plant transformation

Cloning and tissue culture was performed as previously described (Wuddineh et al., 2015). Briefly, the putative homolog of *Arabidopsis* AtSHN2 (At5g11190) and rice OsSHN (Os06g40150) was identified by tblastn or blastp against the switchgrass EST database or draft genome (Phytozome v1.1 DOE-JGI) followed by cluster and multiple sequence alignment analysis to discriminate the most closely related gene for cloning. For construction of overexpression cassette, the open reading frame (ORF) of *PvERF001* was isolated from cDNA obtained from ST1 clonal genotype of ‘Alamo’ switchgrass using gene-specific primers flanking the ORF of the gene and cloned into pANIC-10A expression vector by GATEWAY recombination (Mann et al., 2012). The primer pairs used for cloning are shown in Table S1 in Supplementary Material. Embryogenic callus derived from SA1 clonal genotype of ‘Alamo’ switchgrass (King et al., 2014) was transformed with the expression vector construct through *Agrobacterium*-mediated transformation (Burris et al., 2009). Antibiotic selection was carried out for about 2 months on 30–50 mg/L hygromycin followed by regeneration of orange fluorescent protein reporter-positive callus sections on regeneration medium (Li and Qu, 2011) containing 400 mg/L timentin. Regenerated plants were rooted on MSO medium (Murashige and Skoog, 1962) with 250 mg/L cefotaxime to assure elimination of *Agrobacterium* from the tissues as well as promote shoot regeneration from transgenic callus (Grewal et al., 2006), and the transgenic lines were screened based on the presence of the insert and expression of the transgene. Simultaneously a non-transgenic control line was also generated from callus.

### Plants and growth conditions

T0 transgenic and non-transgenic control plants were grown in growth chambers under standard conditions (16 h⋅day/8 h⋅night light at 24°C, 390 μE⋅m^−2^ s^−1^) and watered three times per week, including weekly nutrient supplements with 100 mg/L Peter’s 20-20-20 fertilizer. Transgenic and non-transgenic control lines were propagated from a single tiller to produce three clonal replicates for measuring growth parameters (Hardin et al., 2013). The plants were grown in 12-L pots in Fafard 3B soil mix (Conrad Fafard, Inc., Agawam, MA, USA) and grown for 4 months to the R1 stage, in which shoot samples were collected to assay the transgene transcript abundance (Moore et al., 1991; Shen et al., 2009). Each sample was snap frozen in liquid nitrogen and macerated with mortar and pestle. The macerated samples were used for RNA extraction as described below.

### RNA extraction and quantitative reverse transcription polymerase chain reaction

RNA extraction and analysis of transgene transcripts were performed as previously described (Wuddineh et al., 2015). Briefly, total RNA was extracted from shoot tip samples of transgenic and non-transgenic control lines using Tri-Reagent (Molecular Research Center, Cincinnati, OH, USA), and 3 μg of the RNA was treated with DNase-I (Promega, Madison, WI, USA). High-Capacity cDNA Reverse Transcription kit (Applied Biosystems, Foster City, CA, USA) was used for the synthesis of first-strand cDNA. Power SYBR Green PCR master mix (Applied Biosystems) was utilized to conduct quantitative reverse transcription polymerase chain reaction (qRT-PCR) analysis according to the manufacturer’s protocol. All the experiments were conducted in triplicates. The list of all primer pairs used for qRT-PCR is shown in Table S1 in Supplementary Material. Analysis of the relative expression was done as previously described (Wuddineh et al., 2015). There was no amplification products observed with all the primer pairs when using only the RNA samples or the water instead of cDNA.

### Determination of leaf water loss

The rate of water loss via leaf epidermal layer was determined as previously described (Zhou et al., 2014). The second fully expanded leaves of both transgenic and non-transgenic plants were excised and soaked in 50 mL distilled water for 2 h in the dark to saturate the leaves. Subsequently, the excess water was removed and initial leaf weight was measured and water loss determined by weighing the leaves every 30 min for at least 3 h. Subsequently, the detached leaves were dried for 24 h at 80°C to determine the final dry weight. The rate of water loss was calculated as the weight of water lost divided by the initial leaf weight.

### Analysis of lignin content and composition

Both qualitative (phloroglucinol–HCl staining) and quantitative [pyrolysis molecular beam mass spectrometry (py-MBMS)] analysis of lignin content was performed as previously described (Wuddineh et al., 2015). Briefly, leaf samples collected at the R1 developmental stage and cleared in a 2:1 solution of ethanol and glacial acetic acid for 5 days were used for staining analysis. The cleared leaf samples were immersed in 1% phloroglucinol (in 2:1 ethanol/HCl) overnight for staining and the pictures were taken at 2× magnification. For the quantification of lignin content and S:G lignin monomer ratio by NREL high-throughput py-MBMS method, tillers were collected at R1 developmental stage, air-dried for 3 weeks at room temperature and milled to 1 mm (20 mesh) particle size. Lignin content and composition were determined on extractives- and starch-free samples (Sykes et al., 2009).

### Determination of sugar release

For analysis of sugar release efficiency, tiller samples at R1 developmental stage were collected and air-dried for 3 weeks at room temperature. The dry samples were pulverized to 1 mm (20 mesh) particle size and sugar release efficiency was determined via NREL high-throughput sugar release assays on extractives- and starch-free samples (Decker et al., 2012). Glucose release and xylose release were measured by colorimetric assays and summed for total sugar release.

### Statistical analysis

To analyze the differences between treatment means, analysis of variance (ANOVA) with least significant difference (LSD) procedure was used while PROC TTEST procedure was used to examine the statistical difference between the expression of target genes in transgenic vs non-transgenic lines using SAS version 9.3 (SAS Institute Inc., Cary, NC, USA). Pearson’s correlation coefficient to determine the relationship between relative transcript levels and growth parameters was calculated by SAS.

## Results

### Identification of AP2/ERF TFs in switchgrass genome

A total of 207 unique switchgrass genes containing one or two AP2 DNA binding domain were identified from the currently available switchgrass EST and genome databases. Amino acid sequence similarities within the conserved AP2 domain between these proteins and previously characterized AP2/ERF TFs from rice and *Arabidopsis* along with the presence of conserved B3 domain suggest that these proteins might be categorized as putative AP2/ERF TFs. The characteristic features of these genes are summarized in Table S2 in Supplementary Material. The amino acid sequences of AP2/ERF TFs showed wide variation in size (ranging from 119 to 666 amino acids) and sequence composition. Twenty-two of these TFs contained two AP2 DNA-binding domains and hence were classified under AP2 family. Five of the AP2/ERF proteins had a B3 conserved domain at the C-terminus in addition to the common AP2 domain, and these genes were grouped into the RAV family. Three of the remaining 180 proteins, namely PvERF049, PvERF160, and PvERF177 with a single AP2 domain, which is more similar to the amino acid sequences of AP2 domains in the AP2 family TFs, were also grouped under the AP2 family. Moreover, one AP2/ERF protein showed a distinct AP2 domain different from all other switchgrass AP2/ERF proteins but with higher shared sequence similarity with the previously identified genes in rice and *Arabidopsis*. The remaining 176 proteins were grouped into ERF family, which was further subdivided into either one of two subfamilies (ERF and DREB) based on sequence similarity in the AP2 domain. The ERF subfamily members included 121 proteins while DREB had only 55 proteins (Table 1).

**Table 1:** **Summary of the AP2/ERF superfamily gene members found in various plant species**.

Family	Subfamily	Group	*Panicum virgatum*	*Oryza sativa*^a^	*Arabidopsis thaliana*^a^	*Populus trichocarpa*^b^
AP2			25	29	18	26
ERF	DREB	I	12	9	10	5
		II	11	15	15	20
		III	27	26	23	35
		IV	5	6	9	6
		Total	55	56	57	66
	ERF	V	10	8	5	10
		VI	9	6	8	11
		VI-L	7	3	4	4
		VII	17	15	5	6
		VIII	25	13	15	17
		IX	37	18	17	42
		X	12	13	8	9
		Xb-L	–	–	3	4
		XI	4	7	–	–
		Total	121	76	65	103
RAV			5	5	6	6
Singleton			1	1	1	1
Total			207	174	147	202

*The switchgrass (*P. virgatum*) data are from this study. Note that switchgrass is the only polyploidy species listed above*.

*^a^Nakano et al. (2006)*.

*^b^Zhuang et al. (2008)*.

The distribution of the identified switchgrass AP2/ERF genes across the nine chromosomes was also evaluated. Thus far, only about half of the switchgrass genomic sequences are mapped into their chromosomal locations based on the draft genome assembly by JGI-DOE available at Phytozome. Accordingly, 166 of the 207 genes could be assigned a chromosomal location. The genes were non-evenly distributed across the nine switchgrass chromosomes wherein the highest number of genes was localized on chromosomes 9, 2, and 1, with the fewest number of genes being assigned to chromosome 8 (Table S3 in Supplementary Material).

### Cluster analysis of switchgrass AP2/ERF proteins

**Figure 1:**
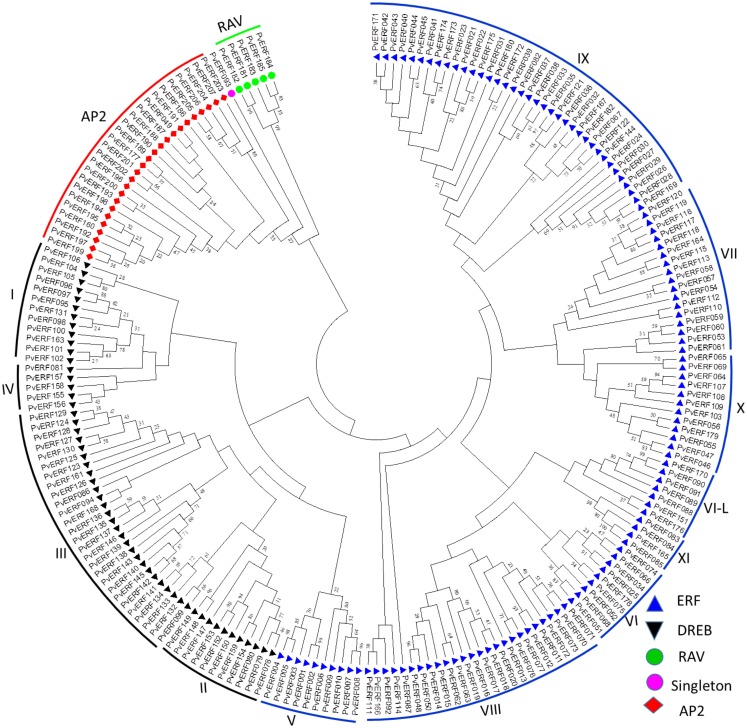
**An unrooted dendrogram of switchgrass AP2/ERF proteins**. The deduced amino acid sequences were imported into MEGA6.0 program and aligned using MUSCLE program. The tree was constructed by a neighbor-joining method with bootstrap replicates of 5000. The families, subfamilies, and groups within each subfamilies are indicated in the tree. The list of switchgrass sequences used to construct this tree along with their gene identifier names are presented in Table S2 in Supplementary Material.

To confirm the classification and evaluate the sequence similarities between the switchgrass AP2/ERF TFs, a dendrogram was constructed by NJ method using the whole amino acid sequences of the proteins. The analysis showed distinct clustering of the proteins into specific groups and families as previously described in other species (Figure 1). Specifically, these clusters highlighted the distinction between the switchgrass AP2, ERF, and RAV families as well as between the ERF and DREB subfamilies. The ERF and DREB subfamilies were further subdivided into seven (groups V–XI) and four (I–IV) distinct groups, respectively. The cluster analysis also resolved the RAV protein family and the singleton into separate clusters, which was in accordance with the sequence similarities in the conserved domains as well as the presence of additional domains in the families/clusters.

### Characterization of AP2/ERF gene structures and conserved motifs

**Figure 2:**
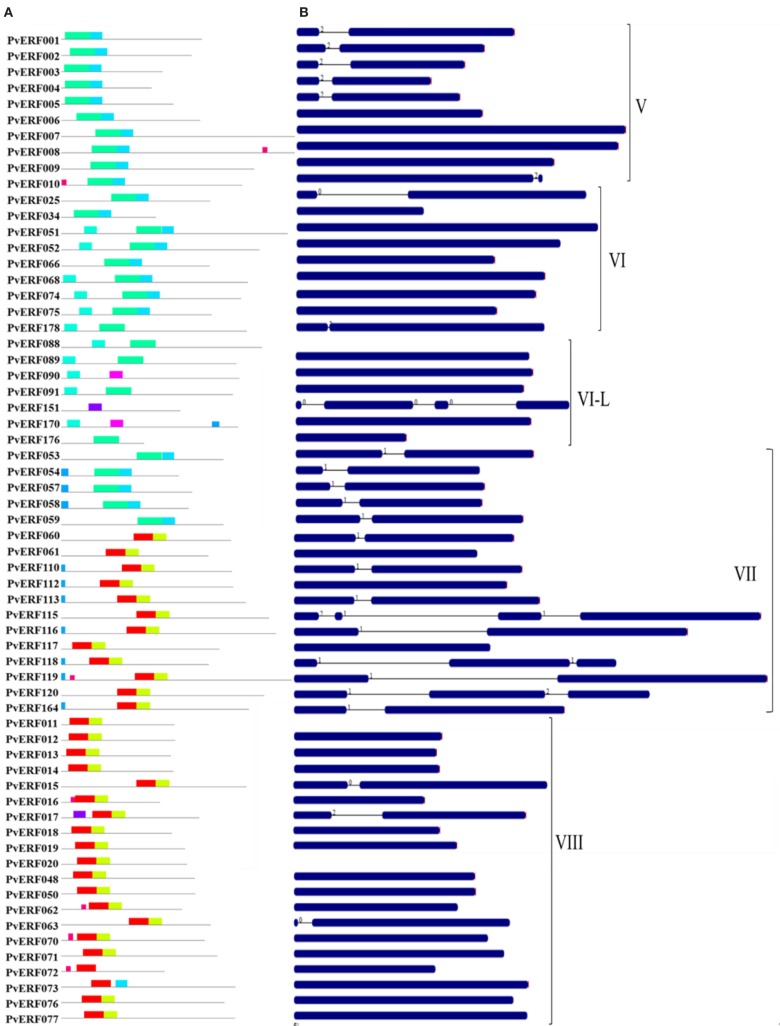

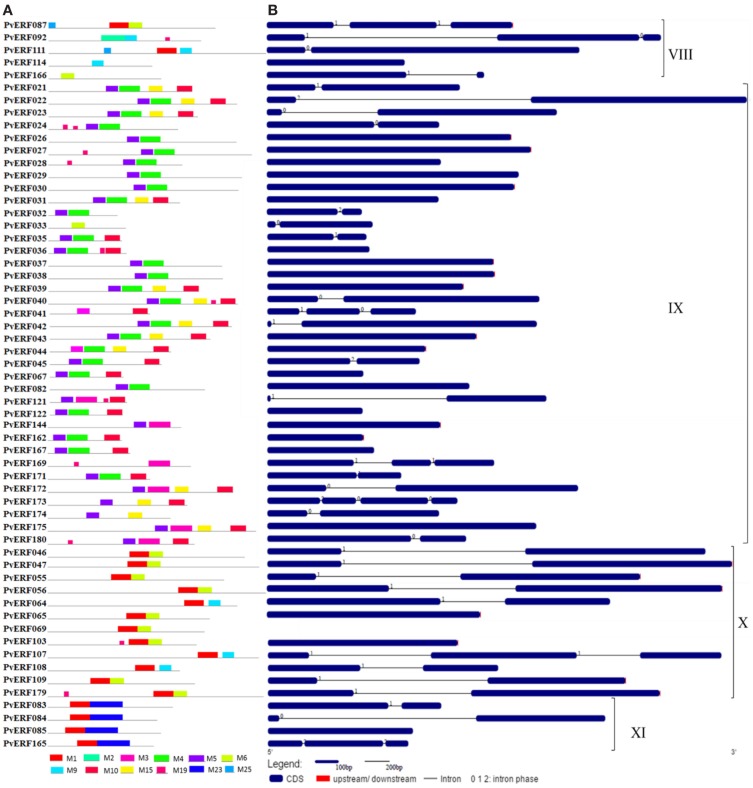
**The schematic representation of protein and gene structures of switchgrass ERF subfamily**. **(A)** Distribution of conserved motifs within the deduced amino acid sequences as determined by MEME tool (Bailey and Elkan, 1994). The colored boxes represent the conserved motifs. **(B)** The gene features as visualized by the gene structure display server (Guo et al., 2007). The coding DNA sequence (CDS) and the untranslated regions (UTR) are shown by filled dark-blue and red boxes, respectively. The introns are shown by thick black lines. The splicing phases of the introns are indicated by numbers. The Roman numerals indicate the group of the genes within the subfamily.

**Figure 3:**
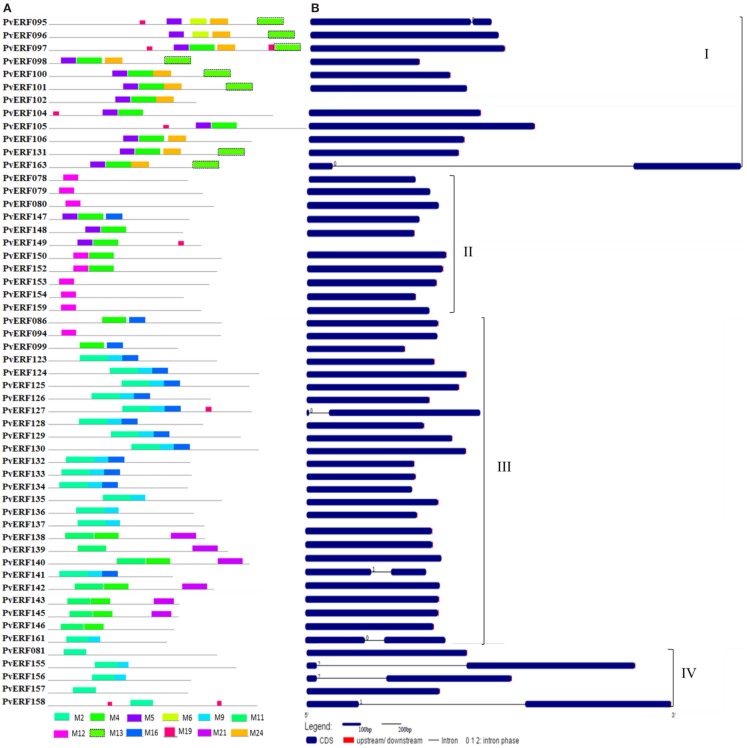
**The schematic representation of protein and gene structures of switchgrass DREB subfamily**. **(A)** Distribution of conserved motifs within the deduced amino acid sequences as determined by MEME tool (Bailey and Elkan, 1994). The colored boxes represent the conserved motifs. **(B)** The gene features as visualized by the gene structure display server (Guo et al., 2007). The coding DNA sequence (CDS) and the untranslated regions (UTR) are shown by filled dark-blue and red boxes, respectively. The introns are shown by thick black lines. The splicing phases of the introns are indicated by numbers. The Roman numerals indicate the group of the genes within the subfamily.

**Figure 4:**
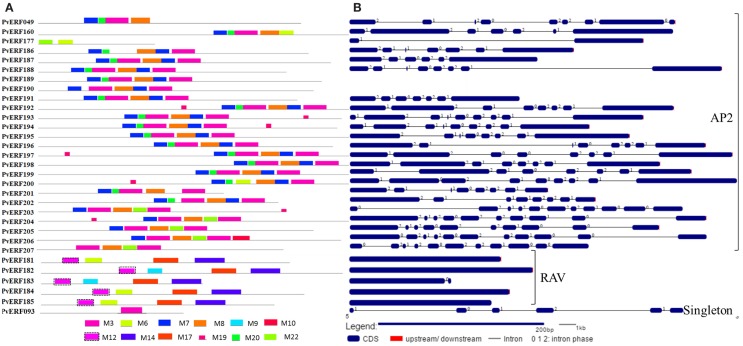
**The schematic representation of protein and gene structures of switchgrass AP2 and RAV families and the singleton**. **(A)** Distribution of conserved motifs within the deduced amino acid sequences as determined by MEME tool (Bailey and Elkan, 1994). The colored boxes represent the conserved motifs. **(B)** The gene features as visualized by the gene structure display server (Guo et al., 2007). The coding DNA sequence (CDS) and the untranslated regions (UTR) are shown by filled dark-blue and red boxes, respectively. The introns are shown by thick black lines. The splicing phases of the introns are indicated by numbers.

To complement the cluster analysis-based classification, the exon–intron structures of AP2/ERF genes were evaluated. The schematic representations of protein and gene structures of switchgrass AP2/ERF superfamily are presented in Figure 2 (ERF), Figure 3 (DREB), and Figure 4 (AP2, RAV, and Singleton). The ORF lengths of these genes vary from 394 bp for the shortest gene to 5409 bp for the longest gene. Analysis of their gene structure showed highly diverse distribution of intron regions within the ORF of the different gene groups or families. The majority of genes belonging to ERF and DREB subfamilies and all but one of the RAV genes appeared to be intronless. Only nine DREB genes (16%) belonging to group I, III, and VI had a single intron in their gene structures. Among ERF genes, 45 (37%) had a single intron in their ORF while eight genes had two and three of them with three introns in its ORF. On the other hand, genes in the AP2 family contained a higher number of introns; ranging from 1 to 10. Only one gene in the AP2 family had a single intron while majority of the genes had more than five introns. The position and state of the introns in the ORF of ERF family genes belonging to groups V, VII, and X show high functional conservation. For instance, about half of the genes belonging to phylogenetic group V in the ERF family showed highly conserved intron positions with an intron phase of two, meaning the location of the intron is found between the second and third nucleotides in the codon. Similarly, the intron positions and splicing phases seems conserved in group VII of the ERF subfamily (Figures 2–4).

Analysis of amino acid sequence conservation in the whole proteins of AP2/ERF superfamily showed the presence of unique conserved motifs shared between proteins within families, subfamilies, or groups (Figures 2–4). Moreover, shared conserved motifs across families, subfamilies, or between groups within subfamilies were also detected, signifying the conservation of the proteins in the AP2/ERF superfamily. In general, a total of 25 conserved motifs (M1–M25) were identified in the superfamily of which 14 motifs, M1–M7, M9, M11, M12, M16, M20, M22, and M23, were related to the AP2 domain (Table S4 in Supplementary Material). The conserved motifs from the non-AP2 domain region appear to specify individual groups within the subfamilies. Among the ERF subfamily, proteins in groups VII and IX have the most diverse set of motifs compared to others while proteins in group XI harbors merely two motifs, M1 and M23 with the last motif being unique to the group (Figure 2). Moreover, shared unique motifs were found in the ERF subfamily proteins belonging to group VII (M25), IX (M10 and M15), VI (M18), and VI-L (M18). Most of the DREB genes belonging to group II have only one specific motif (M12) while a few others have additional motifs such as M5 (Figure 3). The pattern of conserved motif distribution within the largest group in the DREB subfamily (group III) showed the presence of two unique subgroups sharing a set of three conserved motifs, (M2, M9, and M16) and (M4, M11, and M21), respectively. Three of these motifs (11, 16, and 21) were specific to proteins in group III DREB subfamily. DREB subfamily proteins in group I were distinguished by conserved motif-M13 and motif-M24, while group IV DREB genes have unique motif-M2 (Figure 3). Proteins of AP2 family genes harbor four family-specific motifs, namely M7, M8, M20, and M22 (Figure 4). In addition, the majority of AP2 family proteins share M3 with ERF proteins belonging to group IX. Similarly, RAV proteins also possess two unique motifs, M14 and M17 spanning the B3 DNA binding domain, in addition to M6 and M12 spanning the AP2 domain (Figure 4). M6 and M12 motifs are also present in most proteins in the ERF and DREB (group II) subfamilies (Figures 2 and 3; Table S4 in Supplementary Material).

### Gene ontology annotation

**Figure 5:**
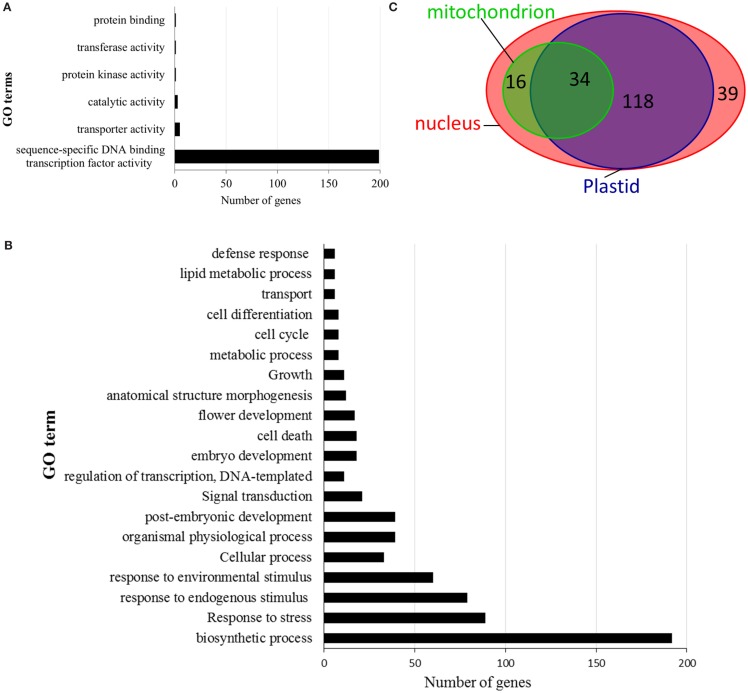
**Summary of the gene ontology (GO) annotation as defined by blast2go**. The switchgrass AP2/ERF genes are categorized according to biological processes **(A)**, molecular function **(B)**, and cellular localization **(C)**.

Gene ontology analysis of switchgrass AP2/ERF TFs, based on rice reference sequences, predicted candidate genes’ molecular functions, putative roles in the regulation of diverse biological processes, and their cellular localization (Figure 5; Table S5 in Supplementary Material). According to blast2GO outputs, over 95% of the switchgrass genes in the AP2/ERF superfamily were predicted to have sequence-specific DNA binding activities (Figure 5A; Table S5 in Supplementary Material). Furthermore, these genes were anticipated to be involved in the regulation of various biosynthetic processes, which could include the biosynthesis of cuticle, waxes, hormones, and other organic compounds. Importantly, many of these genes were also predicted to participate in the regulation of responses to various environmental stresses caused either by biotic factors such as pathogens and insect pests or abiotic factors such as flooding, water deprivation, wounding, and osmotic stress (Figure 5B; Table S5 in Supplementary Material). Cellular localization of the AP2/ERF TFs was predicted by Blast2GO analysis complemented with subcellular localization prediction tool, WoLF PSORT for proteins with heretofore ambiguous results. The results showed that majority of switchgrass AP2/ERF proteins (>80%) were at least dual targeted, i.e., localized to nucleus, plastid, and/or mitochondrion (Figure 5C; Table S5 in Supplementary Material). Only 39 gene products (20%) were predicted to be localized solely to the nucleus (Table S5 in Supplementary Material).

### Expression pattern of switchgrass *AP2/ERF* genes

A switchgrass gene expression atlas (PviGEA) containing expression data for about 78,000 unique transcripts in various tissues was recently developed (Zhang et al., 2013) and is publicly available at web server^7^. To investigate whether the identified switchgrass AP2/ERF genes may have any association with various biological processes that occur during seed germination, vegetative, and reproductive development as well as lignification or cell wall development, transcript data were pooled from the PviGEA web server to assess their expression profile.

**Figure 6:**
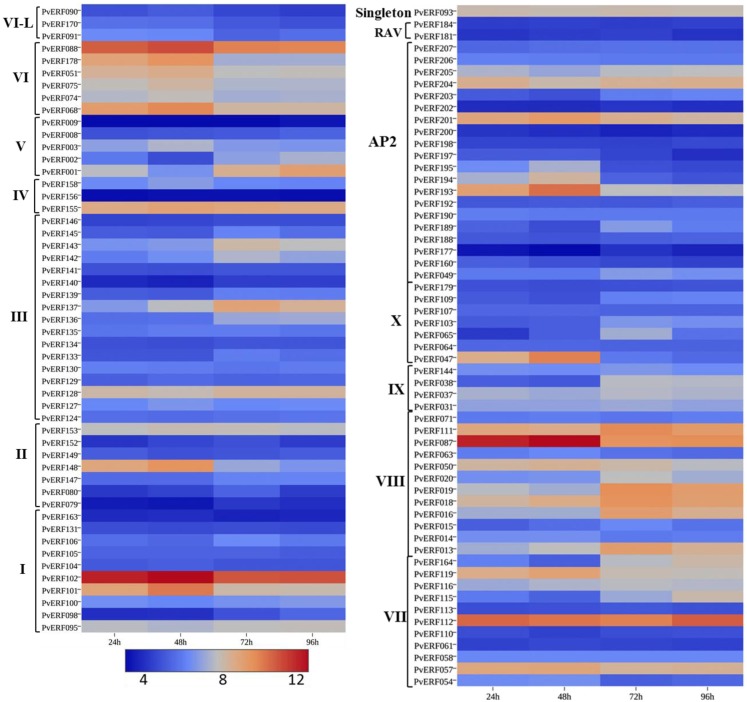
**The expression pattern of putative switchgrass AP2/ERF genes at 24, 48, 72, and 96 h after imbibition**. The heat-map depicting the log2-transformed values of the expression level of each gene was obtained from the switchgrass gene expression atlas (PviGEA). The color scale represents the log2 values of gene expression with blue color denoting low expression and red for high expression. The Roman numerals I–IV represent the groups of the genes in DREB subfamily while V–X showing the groups of genes in ERF the subfamily.

During seed germination (Figure 6; Table S6 in Supplementary Material), some genes in the DREB subfamily showed high expression at early stages of germination (radicle emergence) (48 h after imbibition) while others showed increased expression at later stages of germination (mainly coleoptile emergence) (Figure 6; Table S6 in Supplementary Material). Similarly, the expression of many ERF genes showed dramatic increase during early germination stage while numerous others had peak expression at later stages (coleoptile emergence (72 h) and mesocotyl elongation (96 h) stages. Four of the AP2 family genes (*PvERF193*, *PvERF194*, *PvERF195*, and *PvERF201)* displayed increased expression level at radicle emergence whereas the other two (*PvERF049* and *PvERF203)* showed increased expression at coleoptile emergence. The expression of the RAV genes and the singleton gene were apparently relatively less variable throughout the seed germination process (Figure 6; Table S6 in Supplementary Material).

Comparison of the expression pattern of AP2/ERF genes in roots and shoots at three vegetative phases of development (first, third, or fifth fully collared leaf stages) revealed apparent differential expression pattern between the organs and different stages of vegetative development (Figure S1 and Table S6 in Supplementary Material). Moreover, the expression pattern of AP2/ERF genes during reproductive development also showed differential expression between the reproductive tissues from the initiation of inflorescence meristem to the maturation of the seeds (Figure S2 and Table S6 in Supplementary Material).

### Expression profiles of switchgrass AP2/ERF genes in lignified tissues

**Figure 7:**
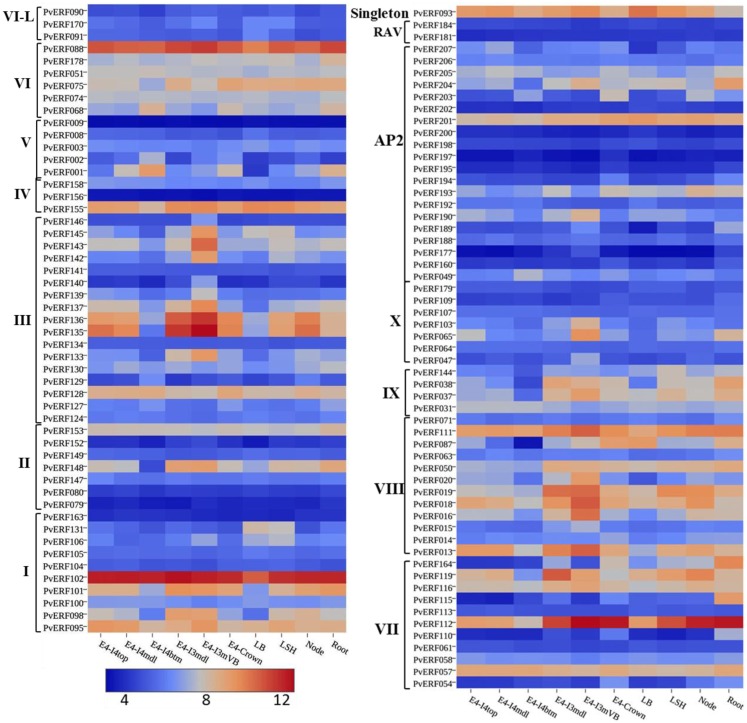
**The expression pattern of putative switchgrass AP2/ERF genes in roots and shoot parts including portions of developing internodes and vascular bundles at stem elongation stage 4 (E4)**. The heat-map depicting the log2-transformed values of the expression level of each gene was obtained from the switchgrass gene expression atlas (PviGEA). The color scale represents the log2 values of gene expression with blue color denoting low expression and red for high expression. The level of expression was reported for roots, nodes, leaf sheath (LSH), leaf blade (LB), whole crown (E4-crown), vascular bundle isolated from fragments of the third internode (E4-I3mVB), middle fragments of the third internode (E4-I3mdl) and from the bottom (E4-I4btm), middle (E4-I4mdl), and top (E4-I4top) fragments of the fourth internode. The Roman numerals I–IV represent the groups of the genes in DREB subfamily while V–X showing the groups of genes in ERF the subfamily.

To evaluate whether the identified switchgrass genes coding for AP2/ERF TFs are associated with the regulation of the cell wall biosynthetic genes during cell wall formation or lignification, the transcripts of the genes extracted from the PviGEA web server were used to compare the level of expression in the lignified tissues of vascular bundles and internode fragments against the expression level in less lignified plant tissues such as LBs and sheath. Four genes in group I (*PvERF95*, *PvERF98*, *PvERF101*, and *PvERF102*) and one gene in group II (*PvERF148*) of the DREB subfamily showed highest expression in vascular bundles and internode tissues followed by internode portions where active lignification is expected (Figure 7; Table S6 in Supplementary Material). The majority of DREB genes belonging to group III were highly expressed mainly in the vascular bundles. Similarly, many genes in the ERF subfamily group VIII (*PvERF013*, *PvERF015*, *PvERF016*, *PvERF018*, *PvERF019*, and *PvERF020*) and X (*PvERF047*, *PvERF065*, and *PvERF103*) showed the highest expression in the vascular bundles followed by youngest internode sections (Figure 7; Table S6 in Supplementary Material). In comparison, only two genes in group IX (*PvERF037* and *PvERF038*), one gene in group VI-L (*PvERF088*), and three genes in group VII (*PvERF111*, *PvERF112*, and *PvERF116*) had high expression in vascular bundles. Contrastingly, some genes in the ERF subfamily belonging to group V (*PvERF001* and *PvERF002*) and VI (*PvERF068*) showed the highest expression in the basal fragments of the fourth internodes (E4) that is under less active lignification. Other genes including *PvERF178* (VI); *PvERF110* (VII), *PvERF115* (VII), and *PvERF164* (VII); and *PvERF038* (IX) had notably high relative expression in roots than in other tissues. Compared to the ERF family genes, the expression of AP2 genes was highly diverse with some genes having high specificity to roots and vascular bundles. The expression of the two RAV genes analyzed was uniformly low throughout whereas the singleton gene was highly expressed in the LBs, LSH as well as the vascular bundles, and young internode sections (Figure 7; Table S6 in Supplementary Material).

### Overexpression of *PvERF001* in switchgrass have enhanced plant growth and sugar release efficiency

**Figure 8:**
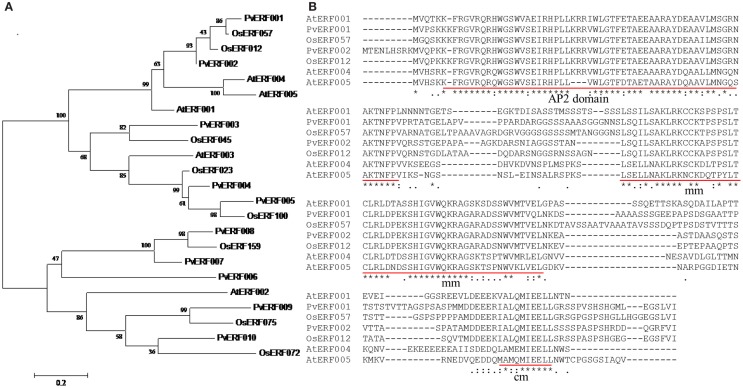
**(A)** Cluster analysis of group V transcription factors in ERF subfamily using the deduced amino acid sequences of switchgrass, rice, and *Arabidopsis*. The sequences were aligned using MUSCLE program and the tree was constructed by maximum likelihood method with Poisson correction and bootstrap values of 1000 in MEGA6.0 program (Tamura et al., 2013). The scale bar shows 0.2 amino acid substitutions per site. **(B)** Multiple amino acid sequence alignment of switchgrass, rice, and *Arabidopsis* ERF TFs. The three conserved motifs that are unique to the rice and *Arabidopsis* ERF TFs are underlined in red. The multiple sequence alignment was constructed using the amino acid sequences of respective genes by MUSCLE program (Edgar, 2004). The locus names of the switchgrass sequences and GenBank accession numbers of the sequences used in this tree are listed in Tables S2 and S7 in Supplementary Material.

**Figure 9:**
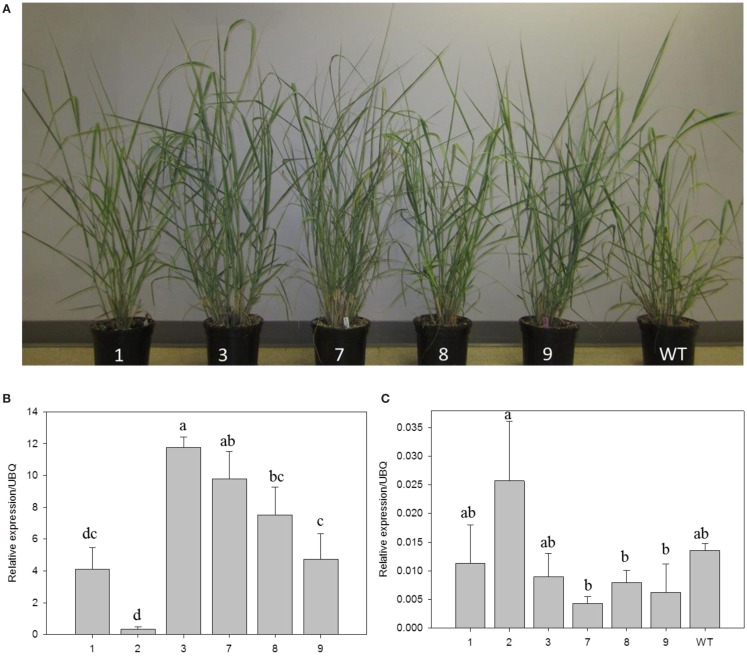
**Representative *PvERF001* overexpressing and non-transgenic control (WT) switchgrass lines (A)**. Relative transcript levels of the transgene **(B)** and endogenous gene **(C)** in *PvERF001* overexpressing and non-transgenic (WT) plants. The expression analysis was done using RNA from the shoot tips at E4 developmental stage. The dissociation curve for the qRT-PCR products showed that the primers were gene-specific. The relative levels of transcripts were normalized to ubiquitin (UBQ). Bars represent mean values of three replicates ±SE. Bars represented by different letters are significantly different at *P* ≤ 0.05 as tested by LSD method with SAS software (SAS Institute Inc.).

Transgenic switchgrass is desired for less recalcitrance biomass for biofuels. To that end, we selected PvERF001, a putative switchgrass homolog of *Arabidopsis* AtERF004 (AtSHN2) and rice OsERF057 (OsSHN) in ERF subfamily group V, for overexpression analysis in switchgrass. This gene was selected since the expression of its *Arabidopsis* homolog in transgenic rice resulted in modified cell wall composition (Ambavaram et al., 2011). Sequence grouping/cluster and sequence alignment analysis suggested that PvERF001 is closely related with its rice and *Arabidopsis* homologs, sharing two highly conserved motifs: the middle motif (mm) and the C-terminal motif (cm) specific to the *Arabidopsis* SHINE clade of TFs (AtERF001, AtERF004, and AtERF005) and OsERF012 and OsERF057 (Figures 8A,B). Thus, the ORF of *PvERF001* was cloned and overexpressed in switchgrass producing more than six independent transgenic lines, which were confirmed based on genomic PCR for the insertion of the transgene and the hygromycin-resistance gene, as well as visualization of OFP in transgenic plants compared to the non-transgenic control lines (Figure 9A; Figures S3A–C in Supplementary Material). Analysis of the transgene expression level by qRT-PCR showed 1–12-fold overexpression in transgenic lines (Figure 9B). The expression of the endogenous gene in transgenic lines was not affected compared to the non-transgenic control line (Figure 9C). All transgenic lines had equivalent or improved vegetative growth metrics relative to the non-transgenic control lines under greenhouse conditions, which was congruent with the relative transcript levels of the transgene [Pearson’s correlation for biomass weights (*R* = 0.77 at *P* < 0.05) and tiller height (*R* = 0.73 at *P* = 0.06)] (Figure 9B; Table 2; Figure S4 in Supplementary Material). Three transgenic lines (3, 7, and 9) had increased biomass. Line 3 had statistically significant increases in four of the six growth traits and approximately twice the dry biomass of the control line (Table 2).

**Table 2:** **Morphology and biomass yields of transgenic switchgrass lines overexpressing *PvERF001* and non-transgenic control (WT) plants**.

Lines	Tiller height (cm)	Tiller number	Fresh weight (g)	Dry weight (g)	Plant diameter (cm)	Fresh/dry weight ratio
1	98.9 ± 2.0^b^	13.3 ± 1.5c^bc^	40.5 ± 1.7d^cd^	12.8 ± 0.3^c^	1.38 ± 0.06^b^	3.15 ± 0.07^a^
2	105.8 ± 2.9b^ab^	12.3 ± 2.6^c^	45.8 ± 11.3^bcd^	15.1 ± 3.9c^bc^	1.36 ± 0.06c^bc^	3.05 ± 0.04^a^
3	115.3 ± 1.2^a^	17.7 ± 1.9b^ab^	70.2 ± 9.9^a^	21.9 ± 3.3^a^	1.54 ± 0.03^a^	3.21 ± 0.08^a^
7	115.7 ± 3.6^a^	21.0 ± 1.6^a^	66.7 ± 3.6b^ab^	17.7 ± 5.5b^ab^	1.23 ± 0.06d^cd^	3.03 ± 0.17^a^
8	101.3 ± 2.1^b^	15.3 ± 0.9^abc^	42.9 ± 1.8d^cd^	13.3 ± 0.9^c^	1.20 ± 0.02^d^	3.23 ± 0.14^a^
9	109.7 ± 3.1b^ab^	17.0 ± 0.8^abc^	61.4 ± 3.6^abc^	16.1 ± 5.2b^ab^	1.44 ± 0.05b^ab^	3.00 ± 0.11^a^
WT	85.8 ± 2.8^c^	15.3 ± 1.5^abc^	34.6 ± 4.7^d^	10.7 ± 1.6^c^	1.05 ± 0.03^e^	3.27 ± 0.08^a^

*Tiller height estimates were determined for each plant by taking the mean of the five tallest tillers within each biological replicate. The fresh and dry biomass measurements were obtained from aboveground plant biomass harvested at similar growth stages. Values are means of three biological replicates ±SEs (*n* = 3). Values represented by different letters are significantly different at *P* ≤ 0.05 as tested by LSD method with SAS software (SAS Institute Inc.)*.

To investigate whether *PvERF001* overexpression could affect the leaf cuticular permeability, the water retention capacity in transgenic and non-transgenic control lines was analyzed in detached leaves measured in the dark to minimize transpirational water loss through stomata. Transgenic lines showed relative reduction in rate of water loss compared with the control lines (Figure S5 in Supplementary Material). However, no tangible difference was observed in the rate of leaf chlorophyll leaching between transgenic and the control lines (data not shown). Subsequently, we analyzed whether the changes in leaf morphology might be accompanied by changes in the expression level of genes in the cutin and wax biosynthesis pathway, in which none were observed (Figure S6 in Supplementary Material). Moreover, overexpression of *PvERF001* in transgenic switchgrass showed relatively reduced expression of some lignin (*PvC4H* and *PvPAL*), hemicellulose (*PvCSLS2*), and cellulose (*PvCESA4*) biosynthetic genes, as well as some of the transcriptional regulators (*PvMYB48*/59 and *PvNST1*) of cell wall biosynthesis (Figures S7A–C in Supplementary Material). The total lignin content in R1 tillers determined by Py-MBMS of cell wall residues and in leaves determined by phloroglucinol–HCl staining did not show sizeable difference between the transgenic and non-transgenic control lines (Figures S8 and S9A in Supplementary Material). Similarly, analysis of the S/G lignin monomer ratio in transgenic lines did not significantly change as compared to that of the non-transgenic control line (Figure S9B in Supplementary Material). However, significant improvement in glucose release efficiency was observed in lines 7 (10%) and 8 (16%) relative to the non-transgenic control line (Table 3). In contrast, none of the transgenic lines released significantly more xylose than the control. The total sugar release, however, was significantly increased in transgenic line 8 by 11% relative to the non-transgenic control (Table 3).

**Table 3:** **Sugar release by enzymatic hydrolysis in transgenic and non-transgenic control (WT) lines**.

Lines	Glucose release (g/g CWR)	Xylose release (g/g CWR)	Total sugar release (g/g CWR)
1	0.214 ± 0.009^d^	0.175 ± 0.003^c^	0.389 ± 0.011^c^
2	0.238 ± 0.008c^bc^	0.182 ± 0.014^abc^	0.420 ± 0.018^b^
3	0.234 ± 0.003^bcd^	0.192 ± 0.008^a^	0.427 ± 0.006b^ab^
7	0.247 ± 0.003b^ab^	0.176 ± 0.009c^bc^	0.423 ± 0.007^b^
8	0.261 ± 0.003^a^	0.188 ± 0.005b^ab^	0.448 ± 0.012^a^
9	0.227 ± 0.020^bcd^	0.188 ± 0.007b^ab^	0.415 ± 0.024^b^
WT	0.225 ± 0.007d^cd^	0.181 ± 0.007^abc^	0.405 ± 0.003c^bc^

*All data are means ± SE (*n* = 3). CWR, cell wall residues. Values represented by different letters are significantly different at *P* ≤ 0.05 as tested by LSD method with SAS software (SAS Institute Inc.)*.

## Discussion

### Significance of AP2/ERF TFs for improvement of bioenergy crops

AP2/ERF TFs constitute one of the largest protein superfamilies in plants. These TFs play a role in regulating a wide array of developmental and growth processes. Thus, they are interesting targets for crop genetic engineering and breeding (Licausi et al., 2013; Bhatia and Bosch, 2014). Numerous TFs belonging to this superfamily have been characterized in various plant species and their potential biotechnological applications in crop improvement has focused primarily on biotic and abiotic stress tolerance (Xu et al., 2011; Licausi et al., 2013; Hoang et al., 2014). However, less effort has been made to utilize this potential for genetic improvement of bioenergy feedstocks such as switchgrass (Bhatia and Bosch, 2014). We found this lack of development to be somewhat anachronistic since these TFs are variably associated with plant growth and cell wall biosynthesis, which are directly related to two most important traits to a bioenergy crops, such as switchgrass: biomass and cell wall recalcitrance.

### Sequence-based classification of putative AP2/ERF TFs in switchgrass

With this in mind, we conducted a whole genome search for putative switchgrass AP2/ERF superfamily of TFs and found 207 members (Figure 1; Table 1; Table S2 in Supplementary Material). Based on comparative genome analysis with the published results in rice, foxtail millet, and *Arabidopsis*, the identified proteins were classified into three families, namely AP2, RAV, and ERF with the later further divided into two subfamilies (ERF and DREB) (Nakano et al., 2006; Lata et al., 2014). The number of genes in the DREB subfamily found in switchgrass (55) was comparable with that of rice (56), *Arabidopsis* (57), and *Populus* (66). All three species along with switchgrass have a singleton in their genome. Consistent with the previous report in rice (Nakano et al., 2006), the switchgrass DREB and ERF subfamilies comprise four and seven groups, respectively. Moreover, based on comparative analysis of the AP2/ERF TFs between different plant species, it seems that group XI of ERF subfamily is specific to monocots as the Xb-L was reported only in dicots (Nakano et al., 2006; Liu et al., 2013). In general, the relative distribution of genes within the different groups in each subfamily appears to be conserved between the three plant species (Table 1). Classification of the switchgrass AP2/ERF TFs into distinct groups was clearly supported by the amino acid sequence-based dendrogram of the identified proteins suggesting robust evolutionary conservation between the superfamily among plant species.

### *In Silico* predicted gene functions and subcellular localization of AP2/ERF TFs in switchgrass

Consistent with the purported role of AP2/ERF proteins as transcriptional regulators of target genes (Magnani et al., 2004), GO analysis predicted that the majority of the switchgrass AP2/ERF genes appear to have DNA-binding activity consistent with the previous observation in foxtail millet (Lata et al., 2014). Therefore, these genes might be associated with the regulation of various biosynthetic processes as well as responses to environmental stimuli as previously demonstrated for numerous genes in other plant species (Xu et al., 2011; Mizoi et al., 2012; Licausi et al., 2013) (Figures 5A,B). The predicted subcellular localization pattern of AP2/ERF superfamily genes in switchgrass, which was mainly to the nucleus as would be expected for transcriptional regulators but also to the plastids and/or mitochondria in addition to the nucleus, was comparable to that reported in foxtail millet (Figure 5C) (Lata et al., 2014). Such multi-localization of the proteins could be attributed to post-translational modifications, protein folding, or interactions with other proteins (Karniely and Pines, 2005), and might serve to facilitate the coordinated regulation of the expression of nuclear and organellar genomes (Duchene and Giege, 2012).

### Gene and protein sequence diversity of switchgrass AP2/ERF TFs

The exon/intron structures of switchgrass *AP2/ERF* genes were analogous with that of foxtail millet (Lata et al., 2014), castor bean (Xu et al., 2013), rice, and *Arabidopsis* (Nakano et al., 2006). Consistent with these species, we observed a high diversity in the distribution of the intron regions of AP2 genes versus a single or no intron in most genes in the ERF and RAV families (Figures 2 and 4). The pattern of intron distribution within the ORF and their splicing phases was highly conserved in genes within specific groups as reported in castor bean (Xu et al., 2013). Consistent with the observation in rice, the majority of proteins in the groups or subfamilies of switchgrass AP2/ERF superfamily could be distinguished by the presence of one or more diagnostic motifs located outside the AP2 domain region (Table S4 in Supplementary Material) (Rashid et al., 2012). These groups or subfamily-specific conservation in gene structures and protein motifs supported the accuracy of the predicted cluster relationships between the switchgrass AP2/ERF TFs.

AP2/ERF TFs that function as repressors or activators of specific target genes are distinguished by the presence of conserved motifs called repression domains (RD) that are highly conserved, or by the presence of activation domains which are generally less conserved (Licausi et al., 2013). One of the characteristic motif in AP2/ERF transcriptional activators is the activation domain, EDLL motif (Tiwari et al., 2012), while repressors have unique RD namely the ERF-associated amphiphilic repression (EAR) motif (LxLxL or DLNxxP) (Kagale and Rozwadowski, 2011) and B3 repression domain (BRD: R/KLFGV) motif (Ikeda and Ohme-Takagi, 2009). Analysis of the switchgrass AP2/ERF TF sequences also indicated the presence of these motifs in many proteins (Table S4 in Supplementary Material). For instance, many genes in group IX of ERF subfamily appear to be transcriptional activators due to the presence of motif M10, which is an EDLL-like motif. Moreover, this motif is rich in acidic amino residues which has been suggested as the characteristics of transcriptional activators (Licausi et al., 2013). Majority of the ERF family TFs in group VIII and DREB family TFs in group I displayed a DLNxxP-like motifs. Four TFs belonging to the AP2 family (PvERF204, PvERF205, PvERF206, and PvERF207) also displayed similar EAR motif while PvERF203 and PvERF207 harbors DLELSL and NLDLS-like RD, respectively. Similarly, switchgrass TFs in RAV family also displayed unique repression domain, RLFGV (Ikeda and Ohme-Takagi, 2009). ERF subfamily TFs in groups VI and VI-L share a characteristic motif at the *N*-terminus (M18), also known as the cytokinin responsive factor (CRF) domain in *Arabidopsis* that is also shared by rice ERF genes belonging to same group in rice ERF subfamily (Nakano et al., 2006). Genes containing the CRF domain (VI and VI-L) were shown to be responsive to cytokinin (Rashotte et al., 2006). The distinguishing *N*-terminal motif in group VII ERF subfamily proteins, M25 was conserved in both *Arabidopsis* and rice as described previously (Nakano et al., 2006). This motif was shown to dictate the stability of proteins based on the level of oxygen via *N*-end rule pathway (Dubouzet et al., 2003; Licausi et al., 2011). DREB genes in rice with characteristic LWSY motif have been shown to function in regulation of drought, cold, and salinity responsive gene expression (Dubouzet et al., 2003). Switchgrass genes belonging to group III in DREB subfamily (*PvERF133*, *PvERF134*, *PvERF135*, *PvERF136*, *PvERF137*, *PvERF139*, *PvERF140*, *PvERF141*, *PvERF142*, *PvERF143*, *PvERF145*, and *PvERF146*) displayed LWSY conserved motif (M21) at the C-terminal and thus may play similar roles. No information is available in the literature on some of the conserved motifs identified here including M8, M13, M14, M15, M17, and M24 (Table S4 in Supplementary Material), which might potentially be specific to switchgrass.

### Diverse expression profiles of switchgrass AP2/ERF TFs and functional implications

Differential expression of genes according to developmental stages and tissue or organ types may provide an insight into the specialized biological processes that are taking place in the specific plant parts (Cassan-Wang et al., 2013; Zhang et al., 2013). The observed pattern of expression for the majority of switchgrass AP2/ERF genes at different stages of plant development as well as in different tissues/organ types highlight the significance of these genes in the regulation of various plant growth and developmental processes at the specific stages (Figures 6 and 7; Figures S1 and S2 in Supplementary Material). One of the engrossing observations in this study is the association of the expression of numerous genes with tissues/organs undergoing lignification or secondary cell wall development/modification, suggesting that these genes may have intrinsic association with the regulatory machinery of cell wall formation/lignification, which is not as well characterized compared to their roles in stress response (Licausi et al., 2013; Bhatia and Bosch, 2014). Activation of genes responsible for cell wall modification has already been reported to be key during the initiation of seed germination in barley (Sreenivasulu et al., 2008; An and Lin, 2011). In agreement with this, we reported here the transcriptional upregulation of ERF (*PvERF057*, *PvERF068, PvERF088*, and *PvERF119*), DREB (*PvERF101, PvERF102*, and *PvERF148*), and AP2 genes (*PvERF193, PvERF201*, and *PvERF204*) during the initiation of seed germination as well as in vascular bundles and internode sections. Moreover, the observed robust expression of 14 DREB, 17 ERF, and 3 AP2 genes in tissues or organs undergoing active lignification (vascular bundles, top or middle internode sections as well as roots) but less robust expression in less lignified tissues (leaves) also supports this assertion (Figure 7). It should also be noted that the transcript levels of several of these genes showed a relative increase with the developmental stage of the plants (Figure 7; Figure S1 in Supplementary Material) while exhibiting only marginal expression in less lignified tissues such as inflorescence meristem and germinating seedlings (Figure 6; Figure S2 in Supplementary Material). Differential gene expression profiling between elongating and non-elongating internodes in maize was used to identify a total of seven AP2/ERF TFs that are highly expressed in non-elongating internodes undergoing secondary wall development suggesting that these genes may involve in the regulation of secondary cell wall formation (Bosch et al., 2011). Moreover, recent study in *Arabidopsis* and rice identified several putative secondary cell wall-related AP2/ERF TFs based on preferential expression in secondary cell wall-related tissues and coexpression analysis (Cassan-Wang et al., 2013; Hirano et al., 2013a; Bhatia and Bosch, 2014). Some of the switchgrass genes identified in this study (PvERF037, PvERF115, PvERF116, PvERF143, PvERF148, and PvERF164) appear to be putative homologs of maize, rice, and *Arabidopsis* genes identified in the aforementioned studies. Overexpression of *Populus* ERF genes in wood-forming tissues of hybrid aspen was recently shown to result in modified stem growth (including increased stem diameter following the overexpression of five different ERF genes), reduced lignification, and enhanced carbohydrate content (cellulose) in the wood of transgenic lines hinting that these TFs may indeed interact with the transcriptional machinery regulating cell wall biosynthesis (Vahala et al., 2013). Another evidence supporting this is a recent study suggesting that an ERF TF from loquat fruit (*Eriobotrya japonica*) (EjAP2-1) is an indirect transcriptional repressor of lignin biosynthesis via interaction with EjMYB1 TFs (Zeng et al., 2015).

### Overexpression of *PvERF001* improved biomass productivity and sugar release efficiency in switchgrass

Based on global gene coexpression analysis, the rice homolog of AtSHN2, OsSHN (OsERF057) was proposed to have a native association with cell wall regulatory and biosynthetic pathways, yet this was not experimentally verified (Ambavaram et al., 2011). In this study, we investigated whether PvERF001, the closest putative switchgrass homolog of these genes based on clustering, sequence alignment analysis, and the sharing of conserved motifs (mm and cm) specific to *Arabidopsis* SHN clade of TFs and the rice SHN, may participate in the regulation of cell wall biosynthesis (Figure 8). Our results suggest that PvERF001 may not be directly involved in the regulation cell wall biosynthesis though its transgenic overexpression resulted in increased sugar release efficiency (Figure S7 in Supplementary Material; Table 3). Despite the observed reduction in relative expression of some lignin biosynthetic genes and their transcriptional regulators in switchgrass that seem to relate with the results in rice overexpressing *AtSHN2*, no significant changes in the lignin content and composition was detected in transgenic switchgrass in contrast to the reduced lignin content observed in rice overexpressing *AtSHN2* (Ambavaram et al., 2011) (Figures S7, S8, and S9A in Supplementary Material). The increased sugar release might be attributed to altered storage carbohydrates such as starches as recently reported in *Arabidopsis* where ectopic expression of rice ERF TF (SUB1A-1) gene resulted in improved enzymatic saccharification efficiency via increased level of starch (Nunez-Lopez et al., 2015). Similar results were obtained from overexpression of maize *corngrass1* microRNA in switchgrass (Chuck et al., 2011). However, whether PvERF001 is associated with starch biosynthesis remains to be determined. Moreover, in contrast to the previous reports where heterologous expression of *AtSHN2* in rice did not significantly affect the growth characteristics of transgenic lines (Ambavaram et al., 2011), overexpression of *PvERF001* resulted in increased plant growth including plant height, stem diameter and aboveground biomass weight in transgenic lines (Table 2). The discrepancy in lignin content and biomass productivity traits between the AtSHN2 and PvERF001 may indicate the differences in functional specialization between the two genes in monocots and dicots even though sequence analysis seems to suggest that they might be homologs. The fact that overexpression of *AtSHN* genes in *Arabidopsis* rather showed association with the regulation of wax, cutin, and pectin biosynthesis supports this assertion (Aharoni et al., 2004; Shi et al., 2011). Moreover, recent study showed that the homolog of *Arabidopsis SHN* genes in tomato (*SlERF52*) was expressed mainly in the abscission zone and functionally associated with the regulation of the pedicel abscission zone-specific transcription of genes including cell wall-hydrolytic enzymes (polygalacturonase and Cellulase) required for abscission (Nakano et al., 2014). These differences in the expression pattern and function may suggest functional divergence between *SlERF52* and its *Arabidopsis* homologs. Functional divergence between homologous TFs in monocots and dicots has also been reported in previous studies involving the homologs of AtMYB58/63, which is a known activator of lignin biosynthesis that did not appear to play similar roles in rice (Hirano et al., 2013b).

A recent study involving overexpression of rice homolog of *AtSHN2*, *OsSHN*, in rice showed enhanced tolerance of transgenic plants to water deprivation and association of the gene with the regulation of wax and cutin biosynthesis and hence named rice wax synthesis regulatory gene (OsWR2) (Zhou et al., 2014). The closest homolog of this gene, OsERF012 (OsWR1), was also shown to be induced by drought stress and involved in the regulation of wax synthesis (Wang et al., 2012). Therefore, we examined whether PvERF001 might be involved in the regulation of wax and cutin biosynthesis. Consistent with previous studies in rice, relative increase in leaf water retention capacity was detected in transgenic plants though the effect on the expression of wax and cutin biosynthetic genes was minimal (Figures S5 and S6 in Supplementary Material). Possible explanation for the observed differences between overexpression of rice and switchgrass homologs might be an indication of the functional divergence in the switchgrass genes due to gene duplication. This may explain the discrepancy between transgenic rice overexpressing rice *SHN* (*OsWR2*) exhibiting reduction in plant height but increase in the number of tillers (Zhou et al., 2014) and transgenic switchgrass overexpressing *PvERF001* showing increased plant height but no difference in number of tillers. This suggests that ERF genes might functionally be highly diversified and PvERF001 may be part of a different pathway than we anticipated such as regulation of responses to biotic stress or other abiotic stress or regulation of cell elongation or division in coordination with the cytokinin pathway, with the latter perhaps explaining the observed increase in biomass and vegetative growth in transgenic lines.

In summary, the expression profiling of the switchgrass AP2/ERF genes provides baseline information as to the putative roles of these genes and thus a useful resource for future reverse genetic studies to characterize genes for economically important bioenergy crops. With the current advancements in switchgrass research and establishment of efficient transformation system, this inventory of genes along with the information provided here could facilitate our understanding regarding the functional roles of AP2/ERF TFs in plant growth and development. Furthermore, it would aid in the identification of potential target genes that may be used to improve stress adaptation, plant productivity, and sugar release efficiency in bioenergy feedstocks such as switchgrass. The increased biomass yield and sugar release efficiency from overexpressing *PvERF001* highlight the potential of these TFs for improvement of bioenergy feedstocks.

## Author Contributions

WW designed and performed the experiments, analyzed the data, and wrote the manuscript. MM participated in experimental design and data analysis, assisted with revisions to the manuscript and coordination of the study. GT, RS, SD, and MD assisted with performing lignin and sugar release assays and contributed in revision of the manuscript. CS conceived the study and its design and coordination, and assisted with revisions to the manuscript. All authors read and consented to the final version of the manuscript.

## Conflict of Interest Statement

The authors declare that the research was conducted in the absence of any commercial or financial relationships that could be construed as a potential conflict of interest.

## Supplementary Material

The Supplementary Material for this article can be found online at http://journal.frontiersin.org/article/10.3389/fbioe.2015.00101

## Footnotes

^1^http://phytozome.jgi.doe.gov/pz/portal.html

^2^http://meme-suite.org/

^3^http://gsds.cbi.pku.edu.cn/

^4^http://www.genscript.com

^5^http://switchgrassgenomics.noble.org/

^6^https://plot.ly/plot

^7^http://switchgrassgenomics.noble.org/
