# Supplementary material for: Identification and Molecular Characterization of the Switchgrass AP2/ERF Transcription Factor Superfamily, and Overexpression of PvERF001 for Improvement of Biomass Characteristics for Biofuel
Source: Front Bioeng Biotechnol. 2015 Jul 20;3:101. doi: 10.3389/fbioe.2015.00101 (PMC4507462; doi:10.3389/fbioe.2015.00101)
Supplement: Supplementary file 1 [file Table_1.DOCX]

**Supplementary Table 1** List of primers used in this study.

| Primer name | Unitranscript ID | Primer sequence | Reference |
| --- | --- | --- | --- |
| Primers for Cloning *PvERF001* | | | |
| PvSHN2F | Pavir.Da00422 | ATGGTGCCGTCGAAGAAGAAGTTC |  |
| PvSHN2R |  | GATGACGAGGCTGCCTTCCAGC |  |
| Primers for genomic DNA PCR | | | |
| Hygro_F |  | TTGCATCTCCCGCCGTTCACAG |  |
| Hygro_R |  | CTGGGGCGTCGGTTTCCACTAT |  |
| 837_Fnew |  | ATGACCGTCCAGCTCAACAAGGA |  |
| 837_R/common |  | ACAGCGACTTCCTGACCATCCT |  |
| Gene-specific primers for qRT-PCR | | | |
| Primers for confirming the overexpression of *PvKN1* | | | |
| F_837 |  | TCTCGCACTCACATGGGATGCTG |  |
| R_AcV5 |  | ACCAGCCGCTCGCATCTTTC |  |
| Gene specific primers for endogenous *PvERF001* gene expression: | | | |
| PvSHN2_F1 | Pavir.Da00422 | ACTCACATGGGATGCTGGAAGG |  |
| PvSHN2_R1 |  | GTGCAACAACCACGCTCTACCA |  |
| Primers for reference gene | | | |
| F_PvUBIQUITIN | AP13CTG25905 | CAGCGAGGGCTCAATAATTCCA | Xu et al., 2011 |
| R_PvUBIQUITIN |  | TCTGGCGGACTACAATATCCA |  |
| Primers for the expression of lignin biosynthesis genes | | | |
| C4H1_1534F | AP13CTG28733 | GGGCAGTTCAGCAACCAGAT | Shen et al., 2012 |
| C4H1_1611R |  | CGCGTTTCCGGGACTCTAG |  |
| PvCOMT_F461 | KanlCTG02872 | CAACCGCGTGTTCAACGA | Shen et al., 2012 |
| PvCOMT_R534 |  | CGGTGTAGAACTCGAGCAGCTT |  |
| 4CL1_1179_F | AP13CTG06049 | CGAGCAGATCATGAAAGGTTACC | Shen et al., 2012 |
| 4CL1_1251_R |  | CAGCCAGCCGTCCTTGTC |  |

**Supplementary Table 1** continued.

| Primer name | Unitranscript ID | Primer sequence | Reference |
| --- | --- | --- | --- |
| PvCCR1. 112_F | AP13ISTG52570 | GCGTCGTGGCTCGTCAA | Shen et al., 2012 |
| PvCCR1. 187_R |  | TCGGGTCATCTGGGTTCCT |  |
| PvCAD_F116 | KanlCTG19538 | TCACATCAAGCATCCACCATCT | Shen et al., 2012 |
| PvCAD_R184 |  | GTTCTCGTGTCCGAGGTGTGT |  |
| PAL_F1 | KanlCTG00004 | CATATAGTGTGCGTGCGTGTGT | Wuddineh et al., 2015 |
| PAL_R1 |  | CTGGCCCGCCAATCG |  |
| C3H_F1 | AP13ISTG41630 | CGTGAACAATGGGATCAGGATAG | Wuddineh et al., 2015 |
| C3H_R1 |  | GCGGACACAACCATCTCAAATAC |  |
| F5H_F1 | AP13ISTG56842 | CCCCGTGCACTGACGATCTAT | Wuddineh et al., 2015 |
| F5H_R1 |  | CCAAGCCAAGGGAAAACACAGTTA |  |
| HCT_973_F | AP13CTG44530 | GCAGAAGGAGCAGCAGTCATC | Shen et al., 2012 |
| HCT_1035_R |  | CGAGCGGCAATAGTCGTTGT |  |
| Primers for cellulose and hemicellulose synthetic genes | | | |
| CESA1_F1 | AP13CTG06092 | GCATCCAGGGTCCAGTTTATGTG |  |
| CESA1_R1 |  | CCAGATCGGCTTCGGTCAATAC |  |
| CESA4_F1 | AP13CTG01684 | GAATGCTCTGGTCCGAGTGTC |  |
| CESA4_R1 |  | TCTGCCAACAGTAGGGTCCATC |  |
| CESA7_F1 | AP13CTG19403 | CTTCTCGCTCGTCTGGGTTAG |  |
| CESA7_R1 |  | AGCTCGATCAATTCAGCACTCG |  |
| CESA9_F1 | AP13CTG17923 | TGATGAGGAGTTCTCGGAGCTG |  |
| CESA9_R1 |  | GCATTAGAAACACCAGCCACCA |  |
| CSLC2_F | AP13CTG06284 | GTAGAAGCAGCCAAGGCACTG |  |
| CSLC2_R |  | GAGTCGCTGTACGCCTCTTG |  |
| CSLD1_F1 | AP13CTG08033 | CACAGCTACCACGTCCACATC |  |
| CSLD1_R1 |  | CGATGACCTTGTCCATGAGGTG |  |

**Supplementary Table 1** continued.

| **Primer name** | **Unitranscript ID** | **Primer sequence** | **Reference** |
| --- | --- | --- | --- |
| **Primers for the expression of wax and cutin biosynthesis genes** | | | |
| CER6_F1 | AP13ISTG69567 | ACCTCGTCCACATCCTCTGCTC |  |
| CER6_R1 |  | CTTGTAGCAGGCGTAGTCCACCA |  |
| CYP86A7-1_F1 | AP13CTG23248 | ACTCGTACAGGTTCGTGGCCTTC |  |
| CYP86A7-1_R1 |  | GGTGAGCGACATCTTCTGCTCCA |  |
| CYP86A7-2_F1 | Pavir.Ea03782.1 | AGACTGTGGACATGCGACGTG |  |
| CYP86A7-2_R1 |  | GGCAACAGGAGCGAACAAACC |  |
| CYP86A7-3_F1 | Pavir.Ib03534.1 | GGAGACAGTGGTTGCGTTTGG |  |
| CYP86A7-3_R1 |  | CTCGTTCCCTCTCGATGGGTGT |  |
| FAE1_F1 | AP13CTG28659 | CTCAACCTCGTCTCCGTGCT |  |
| FAE1_R1 |  | GGAGCATCGCATGAAGGTGTC |  |
| FDH2_F1 | AP13CTG03242 | GTCTCCGTGGCAGAAGATGAGC |  |
| FDH2_R1 |  | CTGCGACGTTCCACTCTCCTTG |  |
| HTH_F1 | Pavir.Ib02493.1 | GTGATCGACAGCTCCACCTTC |  |
| HTH_R1 |  | TCCTCCATCTCTCTGCCTGGA |  |
| KCS1_F2 | AP13ISTG42547 | GACGCTCCTCCCTCCAATACAG |  |
| KCS1_R2 |  | AGCAGCCAGCCTCCATTGTGT |  |
| LACS1_F1 | AP13CTG04675 | GGAGTGCAAGTCGAGGTTGGTG |  |
| LACS1_R1 |  | ACACCCCTTCCCAGTGTGTCTC |  |
