# Supplementary material for: Identification and Molecular Characterization of the Switchgrass AP2/ERF Transcription Factor Superfamily, and Overexpression of PvERF001 for Improvement of Biomass Characteristics for Biofuel
Source: Front Bioeng Biotechnol. 2015 Jul 20;3:101. doi: 10.3389/fbioe.2015.00101 (PMC4507462; doi:10.3389/fbioe.2015.00101)
Supplement: Supplementary file 3 [file Table_3.DOCX]

**Supplementary Table 3** Chromosomal distribution of AP2/ERF superfamily in switchgrass

| **Chromosome** | **DREB** | **ERF** | **RAV** | **AP2** | **Soloist** | **Total** |
| --- | --- | --- | --- | --- | --- | --- |
| 1 | 9 | 11 | - | 2 | 1 | 23 |
| 2 | 5 | 19 | - | 3 | - | 27 |
| 3 | 2 | 5 | 2 | 2 | - | 11 |
| 4 | 3 | 8 | - | 2 | - | 13 |
| 5 | 2 | 9 | 3 | 2 | - | 16 |
| 6 | 3 | 14 | - | 1 | - | 18 |
| 7 | 7 | 11 | - | 2 | - | 20 |
| 8 | - | 7 | - | 1 | - | 8 |
| 9 | 9 | 17 | - | 4 | - | 30 |
| Unmapped | 15 | 20 | - | 6 | - | 41 |
| **Total** | **55** | **121** | **5** | **25** | **1** | **207** |
