# Supplementary material for: Identification and Molecular Characterization of the Switchgrass AP2/ERF Transcription Factor Superfamily, and Overexpression of PvERF001 for Improvement of Biomass Characteristics for Biofuel
Source: Front Bioeng Biotechnol. 2015 Jul 20;3:101. doi: 10.3389/fbioe.2015.00101 (PMC4507462; doi:10.3389/fbioe.2015.00101)
Supplement: Supplementary file 4 [file Table_4.DOCX]

**Supplementary Table 4** Lists of conserved motifs discovered using the MEME Suite version 4.7.0. The height of a letter in the LOGO indicates its relative frequency at the given position.

| Motif | Logo | E value |
| --- | --- | --- |
| [M1](http://nbcr-222.ucsd.edu/opal-jobs/appMEME_4.9.11423847248701-1976198200/meme.html#motif_1) | 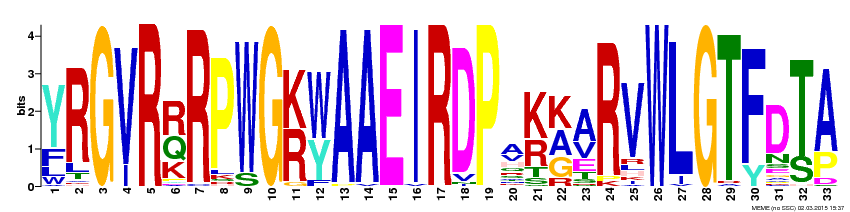 | 1.1e-1189 |
| [M2](http://nbcr-222.ucsd.edu/opal-jobs/appMEME_4.9.11423847248701-1976198200/meme.html#motif_2) | 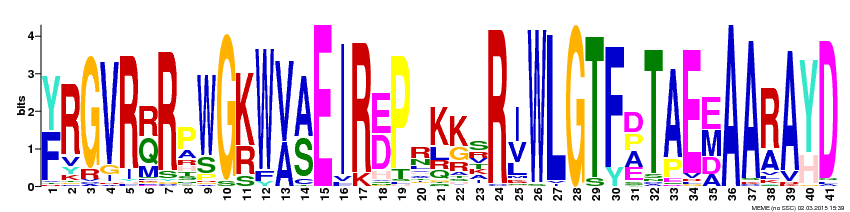 | 6.1e-1323 |
| [M3](http://nbcr-222.ucsd.edu/opal-jobs/appMEME_4.9.11423847248701-1976198200/meme.html#motif_3) | 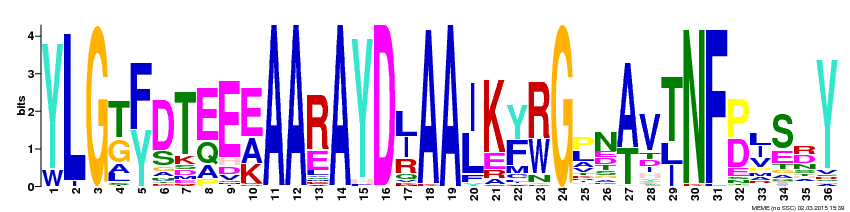 | 5.0e-1057 |
| [M4](http://nbcr-222.ucsd.edu/opal-jobs/appMEME_4.9.11423847248701-1976198200/meme.html#motif_4) | 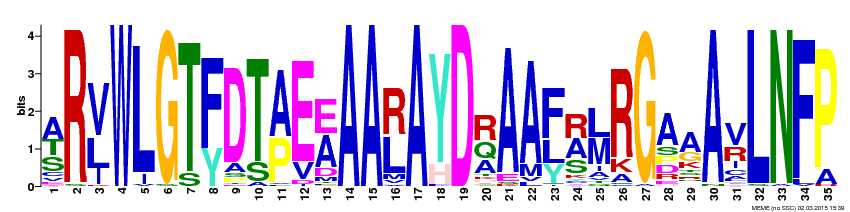 | 3.7e-1048 |
| [M5](http://nbcr-222.ucsd.edu/opal-jobs/appMEME_4.9.11423847248701-1976198200/meme.html#motif_5) | 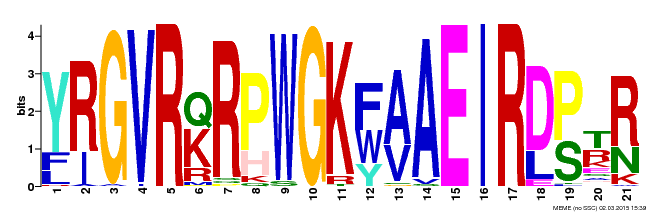 | 3.7e-733 |

**Supplementary Table 4** continued.

| [M6](http://nbcr-222.ucsd.edu/opal-jobs/appMEME_4.9.11423847248701-1976198200/meme.html#motif_6) | 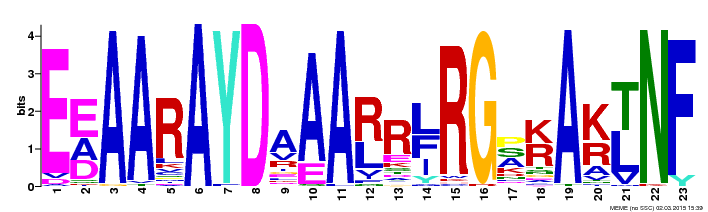 | 5.3e-564 |
| --- | --- | --- |
| [M7](http://nbcr-222.ucsd.edu/opal-jobs/appMEME_4.9.11423847248701-1976198200/meme.html#motif_7) | 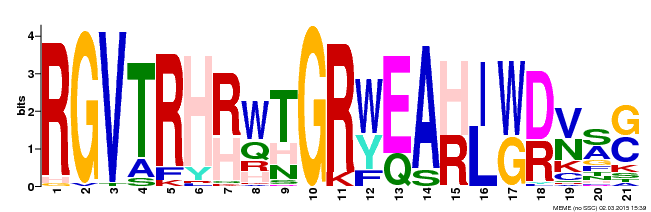 | 1.2e-492 |
| [M8](http://nbcr-222.ucsd.edu/opal-jobs/appMEME_4.9.11423847248701-1976198200/meme.html#motif_8) | 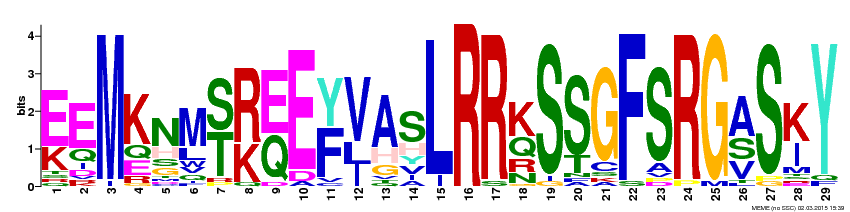 | 1.9e-349 |
| [M9](http://nbcr-222.ucsd.edu/opal-jobs/appMEME_4.9.11423847248701-1976198200/meme.html#motif_9) | 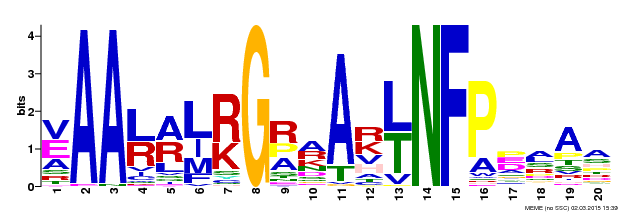 | 7.3e-278 |
| [M10](http://nbcr-222.ucsd.edu/opal-jobs/appMEME_4.9.11423847248701-1976198200/meme.html#motif_10) | 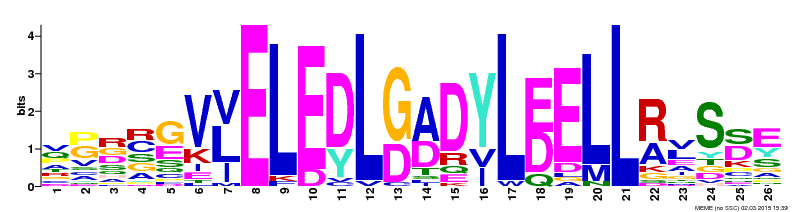 | 6.3e-193 |
| [M11](http://nbcr-222.ucsd.edu/opal-jobs/appMEME_4.9.11423847248701-1976198200/meme.html#motif_11) | 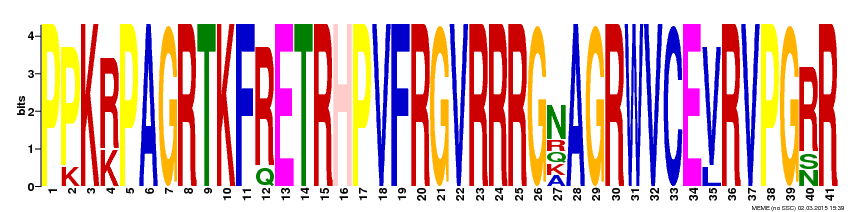 | 4.0e-158 |

**Supplementary Table 4** continued.

| [M12](http://nbcr-222.ucsd.edu/opal-jobs/appMEME_4.9.11423847248701-1976198200/meme.html#motif_12) | 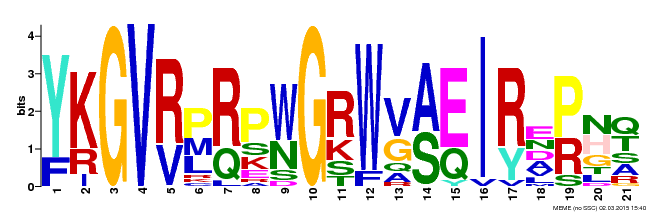 | 7.8e-147 |
| --- | --- | --- |
| [M13](http://nbcr-222.ucsd.edu/opal-jobs/appMEME_4.9.11423847248701-1976198200/meme.html#motif_13) | 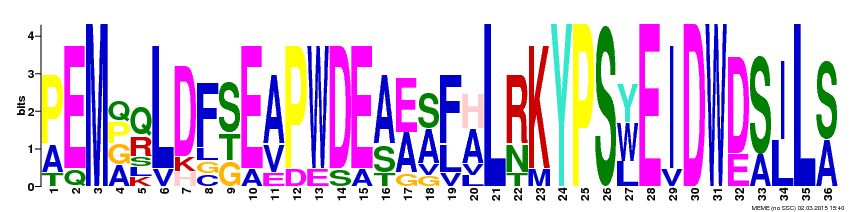 | 1.3e-107 |
| [M14](http://nbcr-222.ucsd.edu/opal-jobs/appMEME_4.9.11423847248701-1976198200/meme.html#motif_14) | 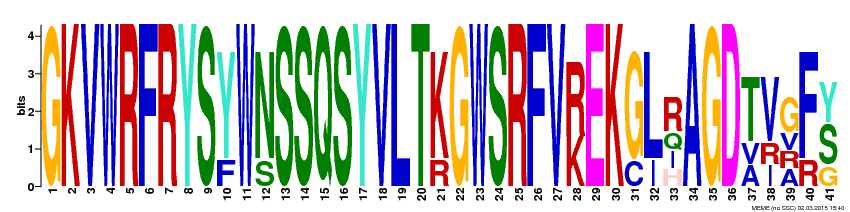 | 2.5e-095 |
| [M15](http://nbcr-222.ucsd.edu/opal-jobs/appMEME_4.9.11423847248701-1976198200/meme.html#motif_15) | 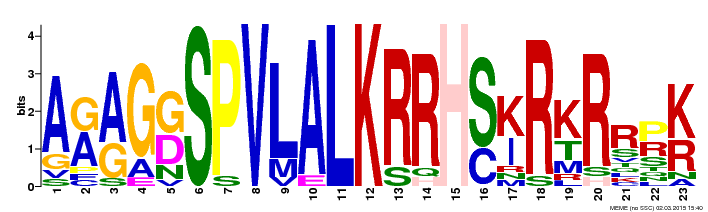 | 1.1e-092 |
| [M16](http://nbcr-222.ucsd.edu/opal-jobs/appMEME_4.9.11423847248701-1976198200/meme.html#motif_16) | 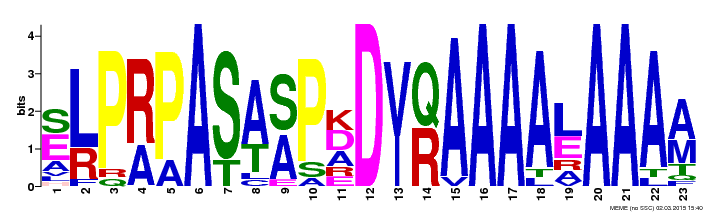 | 8.0e-071 |
| [M17](http://nbcr-222.ucsd.edu/opal-jobs/appMEME_4.9.11423847248701-1976198200/meme.html#motif_17) | 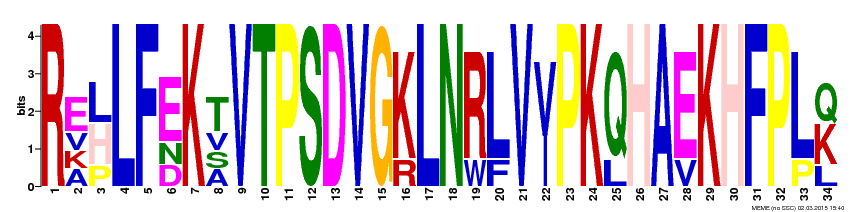 | 1.9e-069 |
| [M18](http://nbcr-222.ucsd.edu/opal-jobs/appMEME_4.9.11423847248701-1976198200/meme.html#motif_18) | 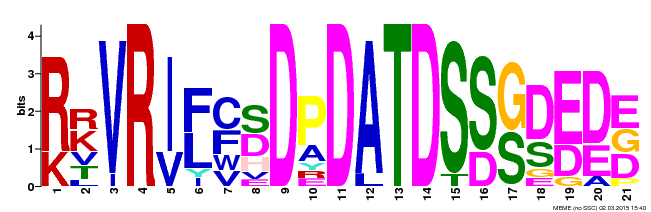 | 7.5e-067 |

**Supplementary Table 4** continued.

| [M19](http://nbcr-222.ucsd.edu/opal-jobs/appMEME_4.9.11423847248701-1976198200/meme.html#motif_19) | 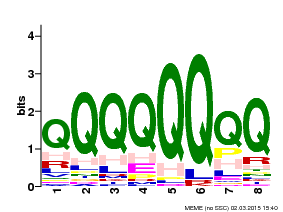 | 1.3e-064 |
| --- | --- | --- |
| [M20](http://nbcr-222.ucsd.edu/opal-jobs/appMEME_4.9.11423847248701-1976198200/meme.html#motif_20) | 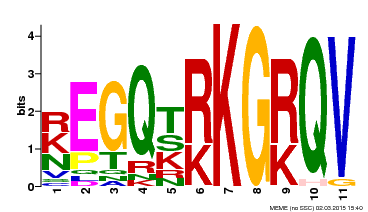 | 2.9e-063 |
| [M21](http://nbcr-222.ucsd.edu/opal-jobs/appMEME_4.9.11423847248701-1976198200/meme.html#motif_21) | 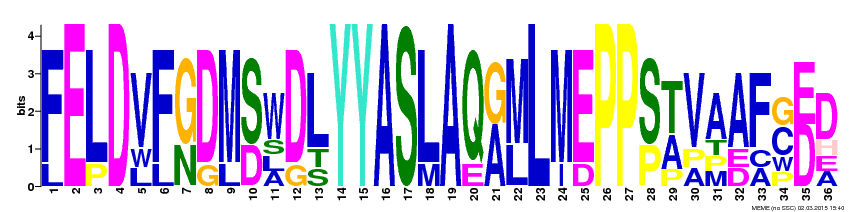 | 1.4e-060 |
| [M22](http://nbcr-222.ucsd.edu/opal-jobs/appMEME_4.9.11423847248701-1976198200/meme.html#motif_22) | 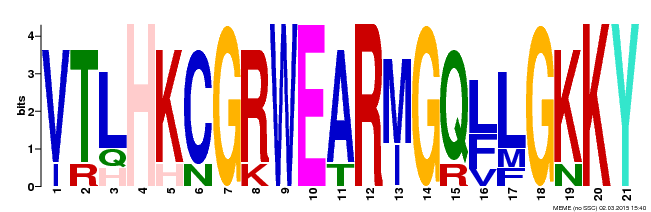 | 5.3e-058 |
| [M23](http://nbcr-222.ucsd.edu/opal-jobs/appMEME_4.9.11423847248701-1976198200/meme.html#motif_23) | 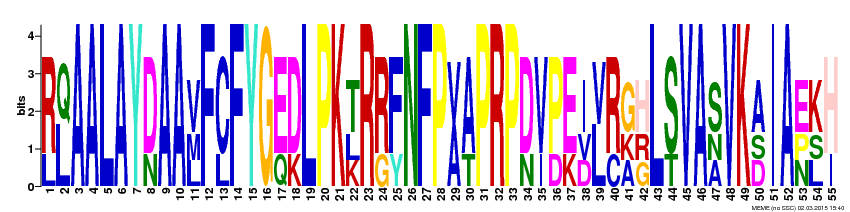 | 9.8e-054 |
| [M24](http://nbcr-222.ucsd.edu/opal-jobs/appMEME_4.9.11423847248701-1976198200/meme.html#motif_24) | 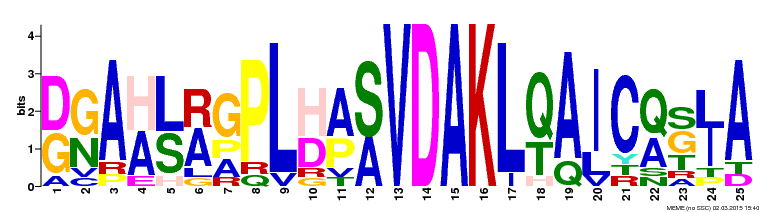 | 1.4e-053 |
| [M25](http://nbcr-222.ucsd.edu/opal-jobs/appMEME_4.9.11423847248701-1976198200/meme.html#motif_25) | 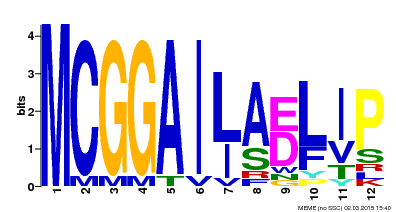 | 1.4e-050 |
