# Supplementary material for: Identification and Molecular Characterization of the Switchgrass AP2/ERF Transcription Factor Superfamily, and Overexpression of PvERF001 for Improvement of Biomass Characteristics for Biofuel
Source: Front Bioeng Biotechnol. 2015 Jul 20;3:101. doi: 10.3389/fbioe.2015.00101 (PMC4507462; doi:10.3389/fbioe.2015.00101)
Supplement: Supplementary file 7 [file Table_7.DOCX]

**Supplementary Table 7** List of the locus names and/or GenBank accession numbers of the sequences used in Figure 8.

| Gene name | MSU Locus name/accession number | Species |
| --- | --- | --- |
| OsERF012 | LOC_Os02g10760.1 | *Oryza sativa* |
| OsERF023 | LOC_Os02g55380.1 | *Oryza sativa* |
| OsERF045 | LOC_Os04g56150.1 | *Oryza sativa* |
| OsERF057 | LOC_Os06g40150.1 | *Oryza sativa* |
| OsERF072 | LOC_Os07g10410.1 | *Oryza sativa* |
| OsERF075 | LOC_Os07g38750.1 | *Oryza sativa* |
| OsERF100 | LOC_Os06g08340.1 | *Oryza sativa* |
| OsERF159 | LOC_Os12g39330.1 | *Oryza sativa* |
| AtERF001 | At1g15360 | *Arabidopsis thaliana* |
| AtERF002 | At5g19790 | *Arabidopsis thaliana* |
| AtERF003 | At5g25190 | *Arabidopsis thaliana* |
| AtERF004 | At5g11190 | *Arabidopsis thaliana* |
| AtERF005 | At5g25390 | *Arabidopsis thaliana* |
