# Supplementary material for: Identification and Molecular Characterization of the Switchgrass AP2/ERF Transcription Factor Superfamily, and Overexpression of PvERF001 for Improvement of Biomass Characteristics for Biofuel
Source: Front Bioeng Biotechnol. 2015 Jul 20;3:101. doi: 10.3389/fbioe.2015.00101 (PMC4507462; doi:10.3389/fbioe.2015.00101)
Supplement: Supplementary file 8 [file Presentation_1.PDF]

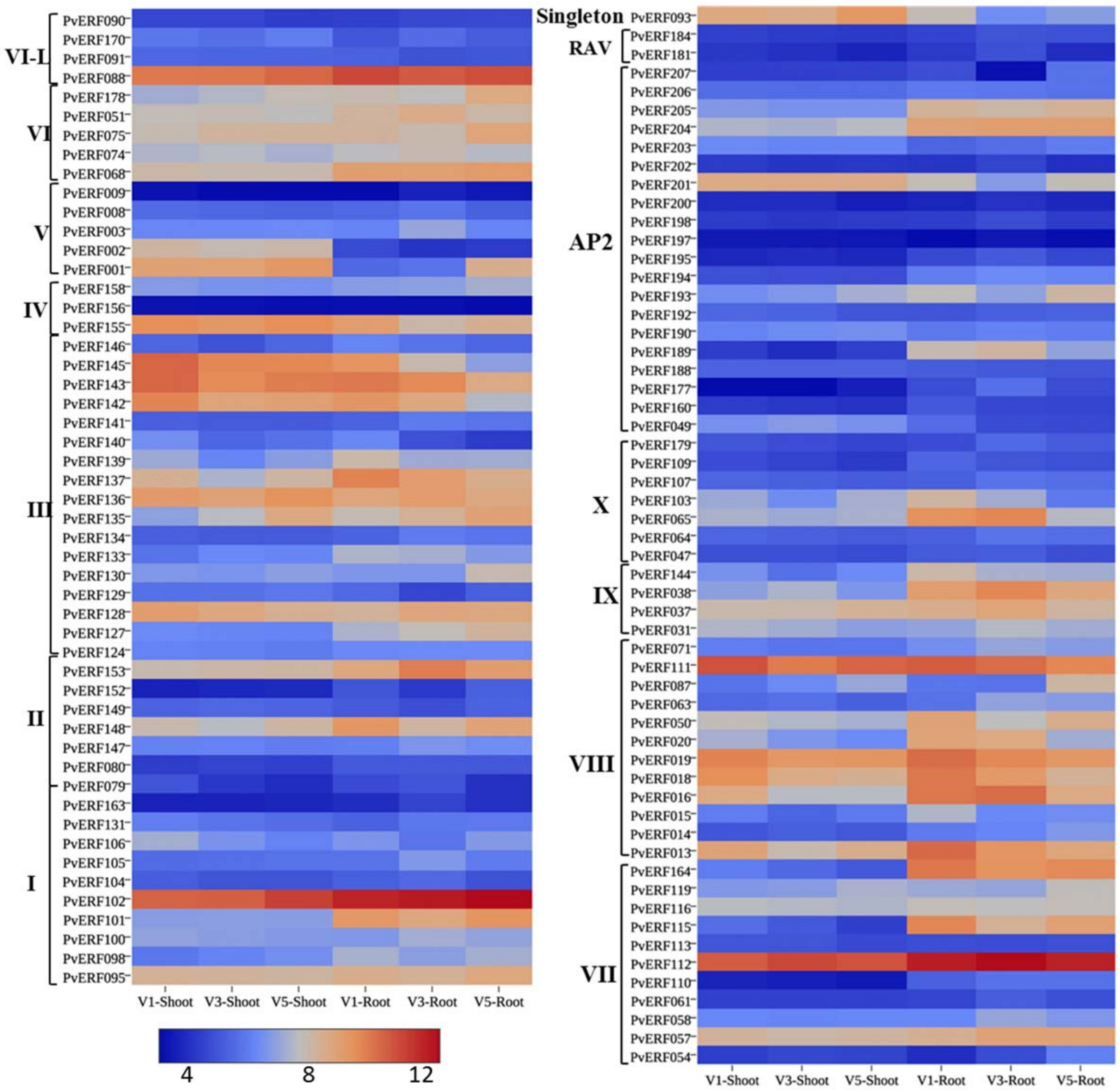

Supplementary Figure 1 The expression pattern of putative switchgrass AP2/ERF genes in roots and shoots during vegetative development. The heat-map depicting the log2 transformed values of expression level of each gene was obtained from the switchgrass gene expression atlas (PviGEA). The color scale represents the log2 values of gene expression with blue color denoting low expression and red for high expression. V1 to V5 represents the vegetative developmental stages from first fully collared leaf stage to the fifth leaf stage when the level of expression both in root and shoot samples was determined. The Roman numerals I-IV represent the groups of the genes in DREB subfamily while V-X showing the groups of genes in ERF the subfamily.

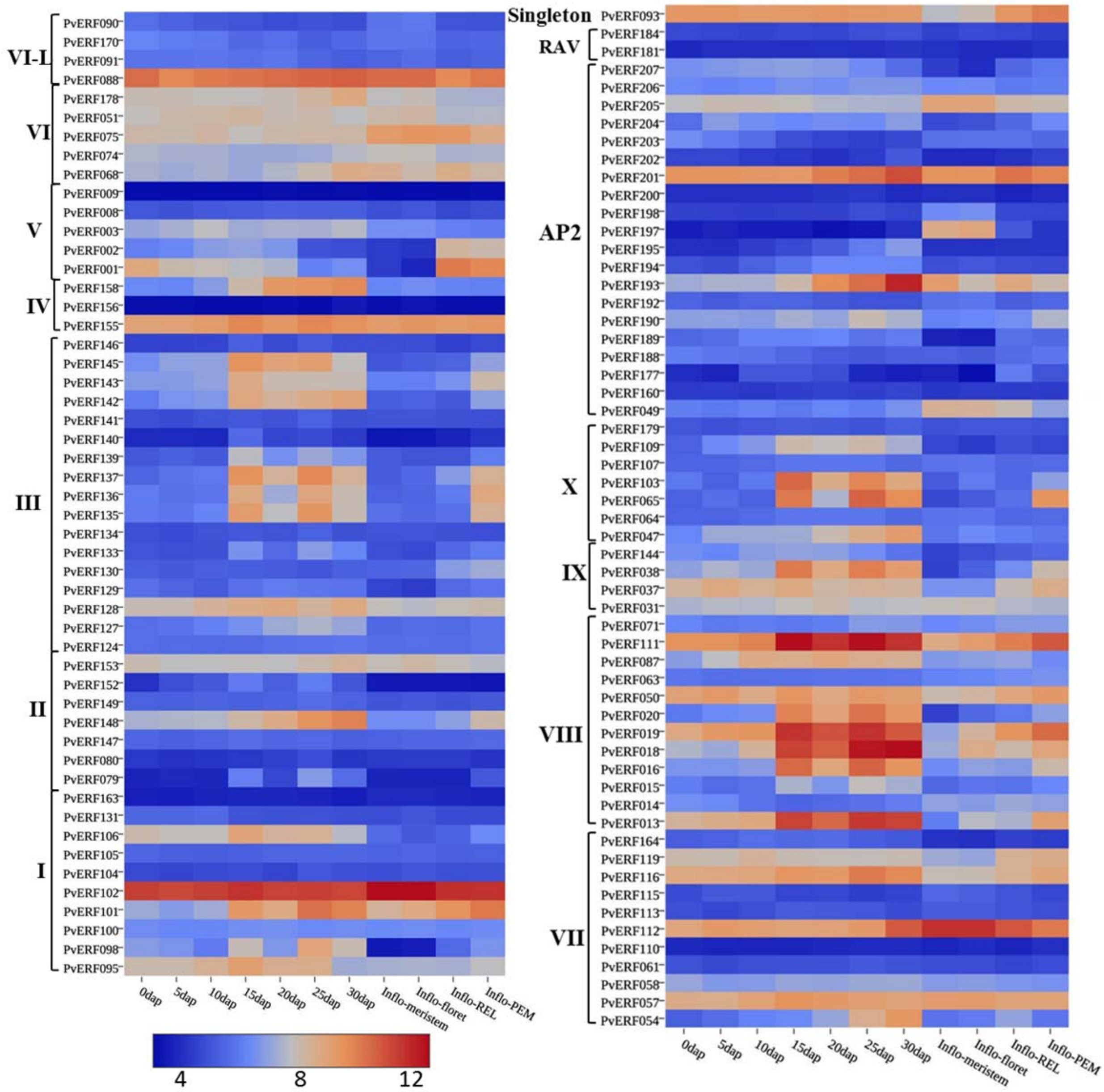

Supplementary Figure 2 The expression pattern of putative switchgrass AP2/ERF genes during reproductive development from floret meristem initiation to mature seed development. The heat-map depicting the log2 transformed values of the expression level of each gene was obtained from the switchgrass gene expression atlas (PviGEA). The color scale represents the log2 values of gene expression with blue color denoting low expression and red for high expression. The expression level was shown for developmental stages of inflorescence including meristem initiation (inflo-meristem), floret development (inflo-floret), rachis and inflorescence branch elongation (inflo-REL) and panicle emergence (inflo-PEM), and pollination and seed development from anthesis (0 days after pollination (dap)) to physiological maturity (30 dap). The Roman numerals I-IV represent the groups of the genes in DREB subfamily while V-X showing the groups of genes in ERF the subfamily.

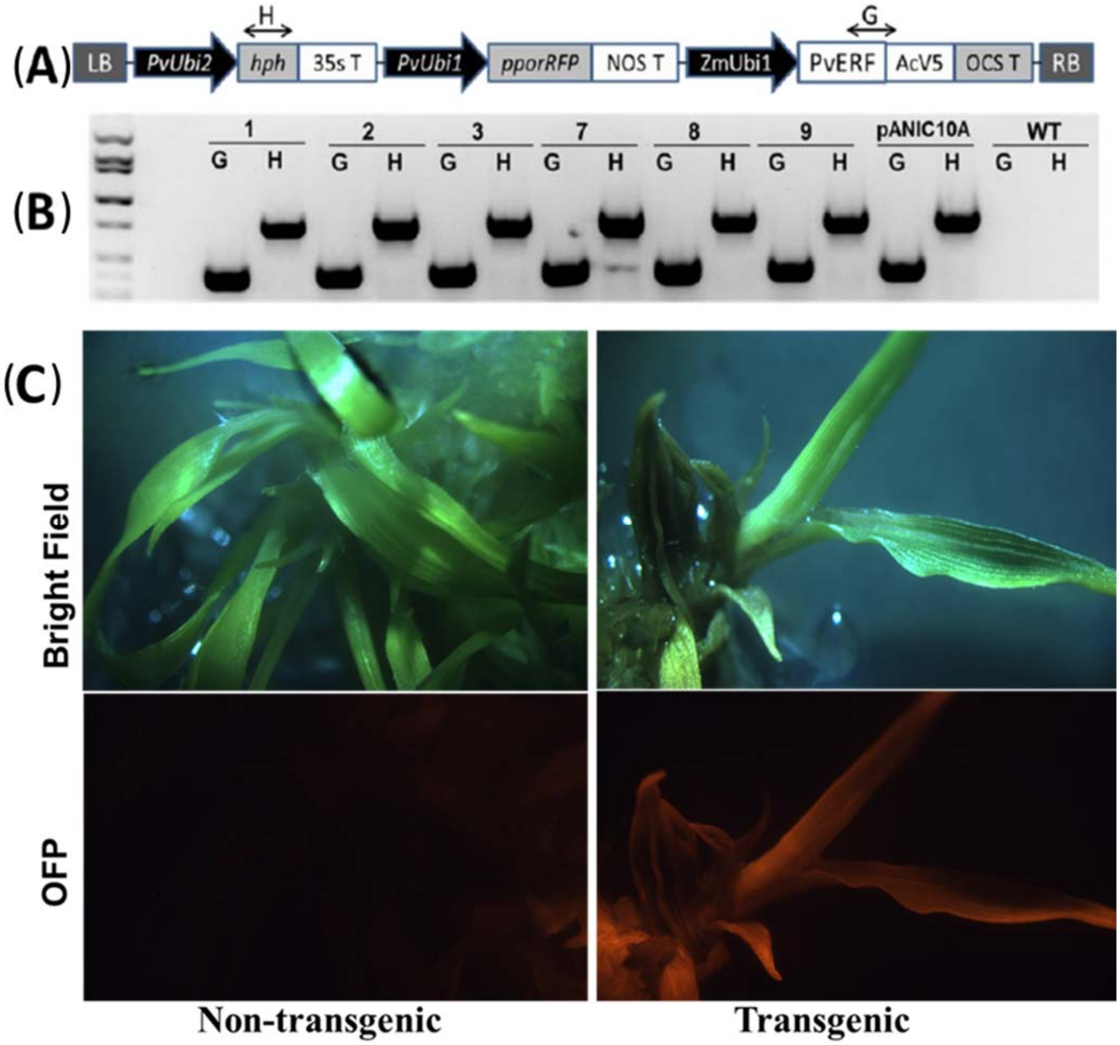

Supplementary Figure 3 Molecular characterization of transgenic switchgrass plants overexpressing the *PvERF001* gene. A) pANIC10A vector construct used for overexpression of *PvERF001*. B) Genomic PCR confirming the insertion of the transgene (G) and the hygromycin-resistance (H) genes in transgenic lines. No amplification was observed in DNA samples from the non-transgenic (WT) plants. C) Orange fluorescence protein (*pporRFP*; OFP) visualization in transgenic plants compared to the non-transgenic control.

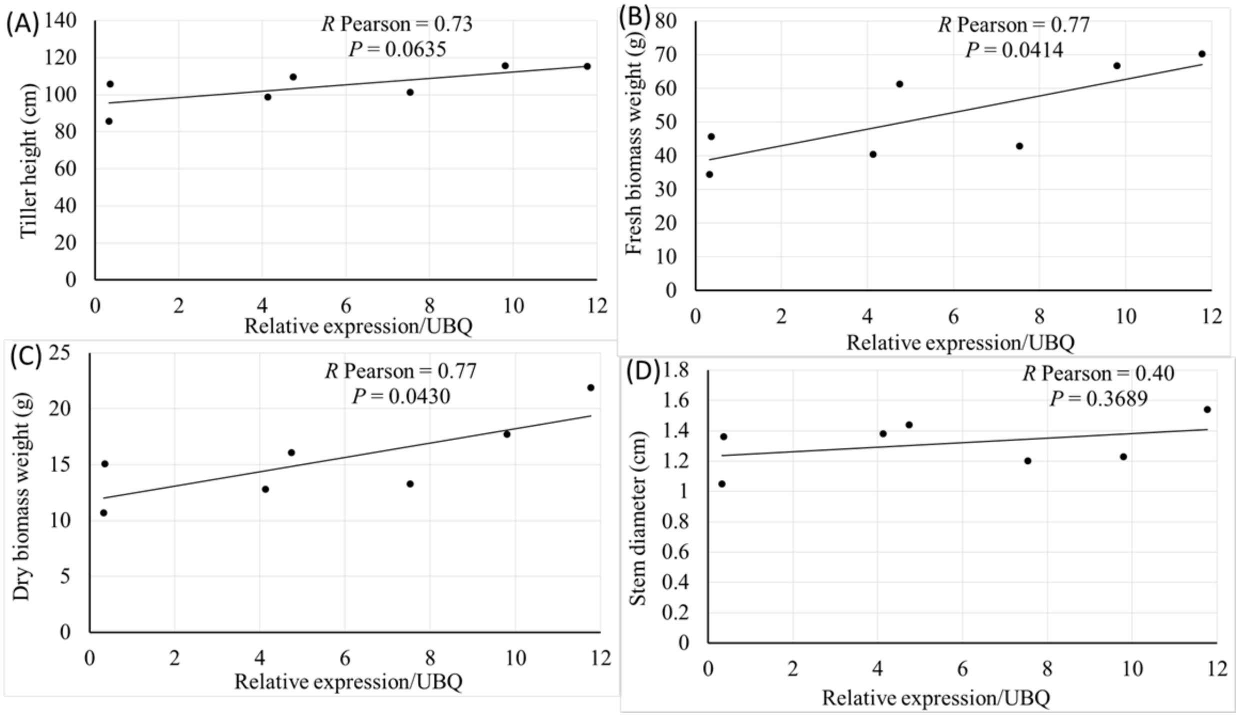

Supplementary Figure 4 Correlation between relative transcript levels and growth metrics: tiller height (A), fresh biomass weight (B), dry biomass weight (C) and stem diameter (D) in switchgrass lines.

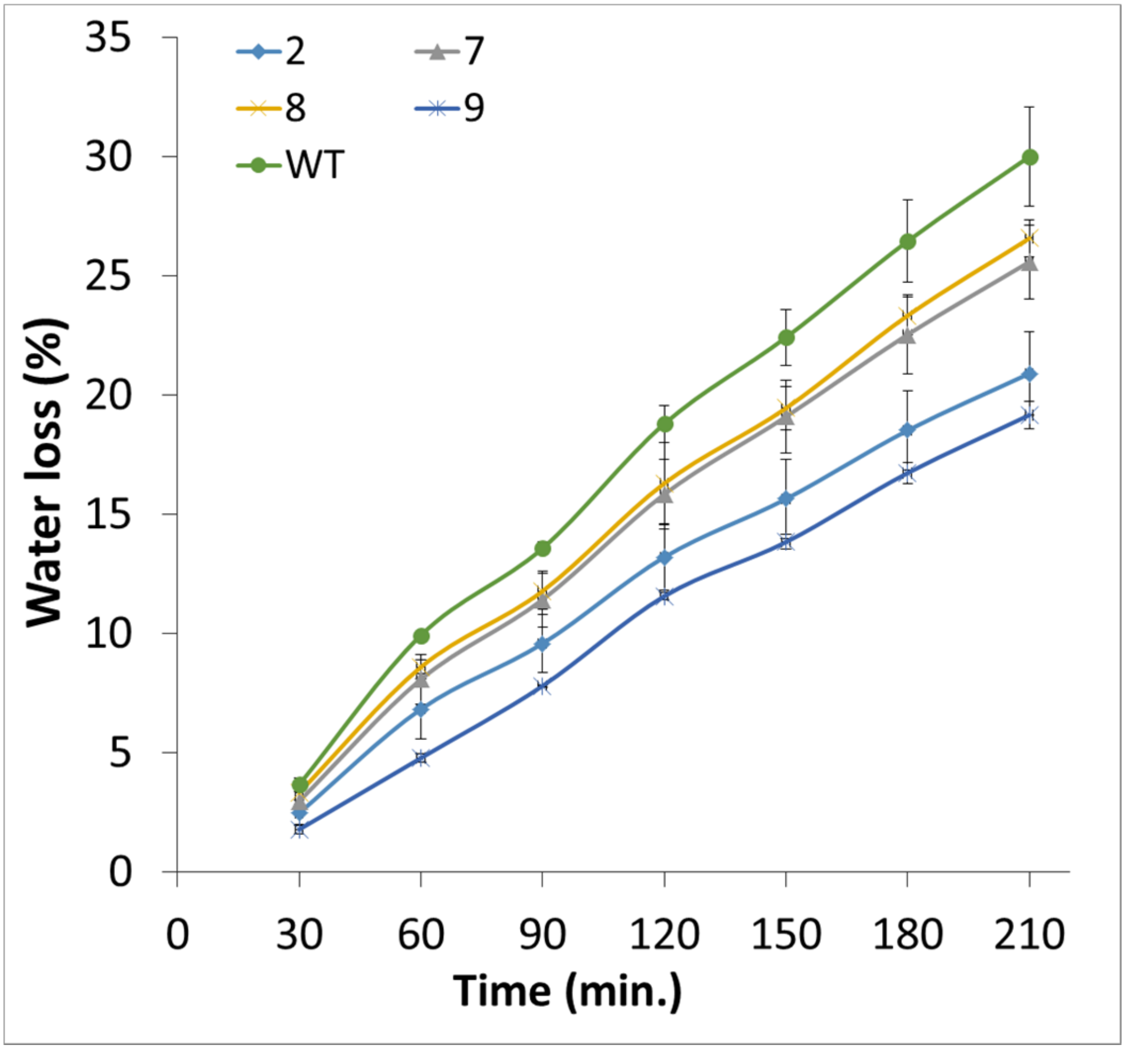

Supplementary Figure 5 Comparison of the relative rate of water loss between the transgenic and non-transgenic control lines. The data points indicated are means of three replicates. Error bars represent mean ± SD.

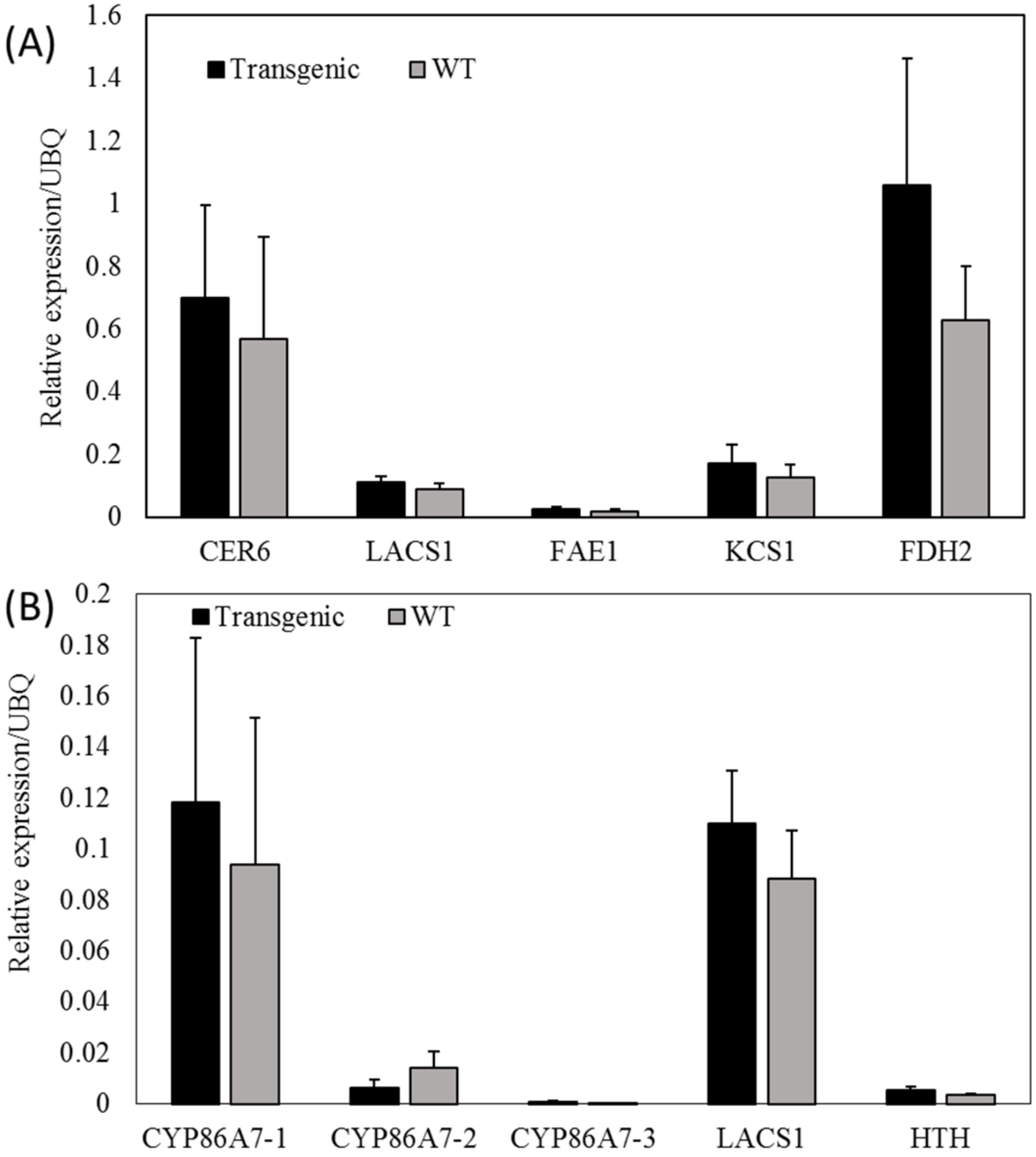

Supplementary Figure 6 The relative expression of putative target genes of *PvERF001* in transgenic vs the non-transgenic (WT) plants as determined by qRT-PCR. A) Wax biosynthetic genes B) Cutin biosynthetic genes. The relative levels of transcripts were normalized to ubiquitin (UBQ). Bars represent mean values of 3 replicates ± standard error. Asterisks indicate significant differences from non-transgenic control plants at P ≤0.05 as determined by PROC TTEST procedure using SAS software (SAS Institute Inc.). *LACS1* (Long-chain acyl-CoA synthase 1); *CER6* (Eceriferum 6); *FAE1* (fatty acid elongase1); *KCS1* (3-ketoacyl-CoA synthase 1); *FDH2* (Formate dehydrogenase 2); *CYP86A7-1* (Cytochrome P450); *HTH* (HOTHEAD protein).

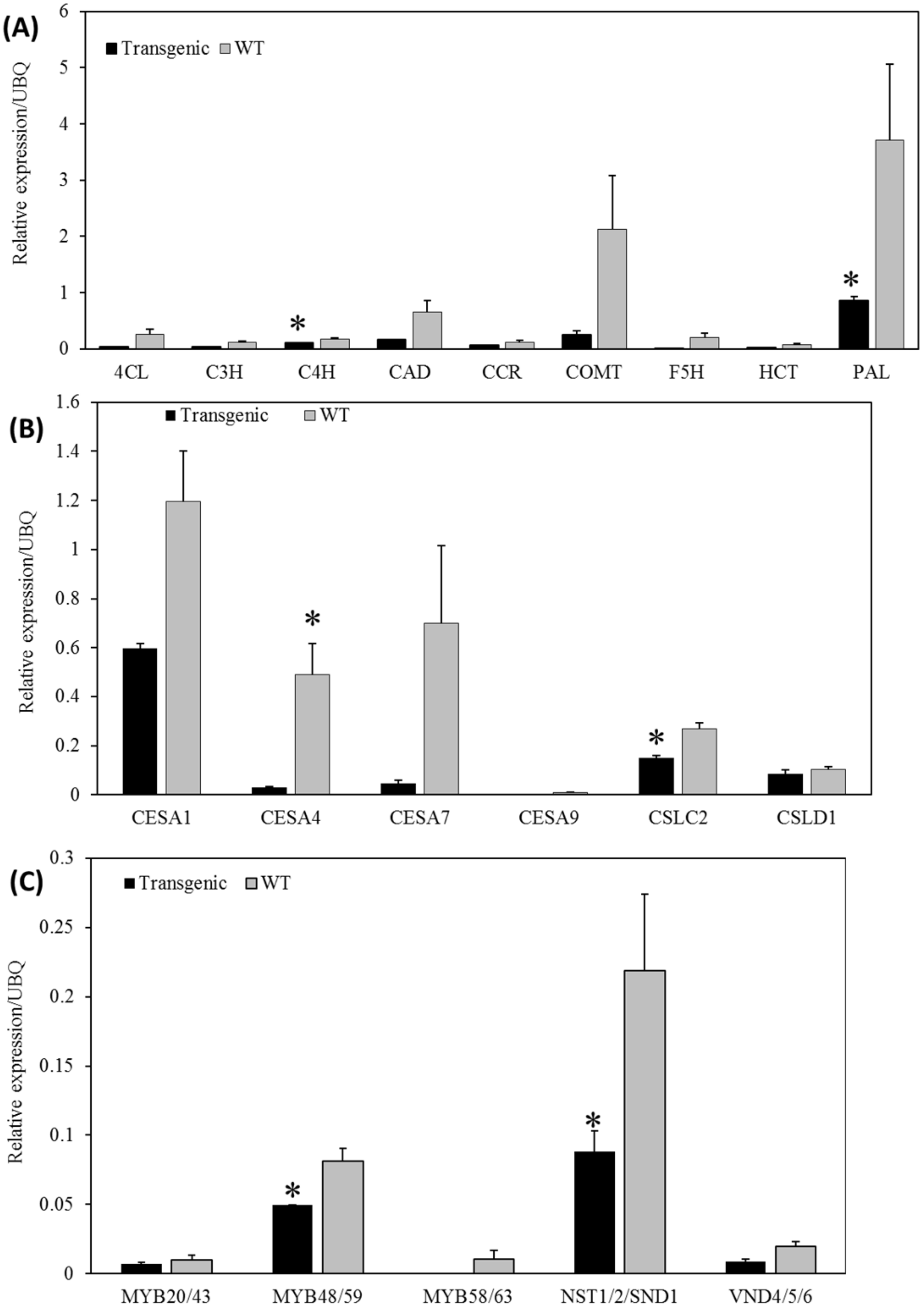

(A)
Transgenic
WT
Relative expression/UBQ
0
1
2
3
4
5
6
*
*
4CL
C3H
C4H
CAD
CCR
COMT
F5H
HCT
PAL
(B)
Transgenic
WT
Relative expression/UBQ
0
0.2
0.4
0.6
0.8
1
1.2
1.4
1.6
*
*
CESA1
CESA4
CESA7
CESA9
CSLC2
CSLD1
(C)
Transgenic
WT
Relative expression/UBQ
0
0.05
0.1
0.15
0.2
0.25
0.3
*
*
MYB20/43
MYB48/59
MYB58/63
NST1/2/SND1
VND4/5/6

Supplementary Figure 7 The relative expression of putative target genes of *PvERF001* in transgenic vs the non-transgenic (WT) plants as determined by qRT-PCR. A) Lignin biosynthetic genes B) Cellulose and hemicellulose biosynthetic genes. C) Transcriptional regulators of cell wall biosynthetic genes. The relative levels of transcripts were normalized to ubiquitin (UBQ). Bars represent mean values of 3 replicates ± standard error. Asterisks indicate significant differences from non-transgenic control plants at $P \leq 0.05$ as determined by PROC TTEST procedure using SAS software (SAS Institute Inc.). *4CL* (4-coumarate: CoA ligase); *C3H* (coumaroyl shikimate 3-hydroxylase); *C4H* (coumaroyl shikimate 4-hydroxylase); *CAD* (cinnamyl alcohol dehydrogenase); *CCR* (cinnamoyl CoA reductase); *COMT* (caffeic acid 3-O-methyltransferase); *F5H* (ferulate 5-hydroxylase); *PAL* (phenylalanine ammonia-lyase); *CESA* (cellulose synthase); *CSL* (cellulose synthase-like). The cellulose and hemicellulose biosynthetic genes as well as the cell wall related TFs were labeled according to the naming from the closest rice or *Arabidopsis* homologs used in Ambavaram *et al*. (2011).

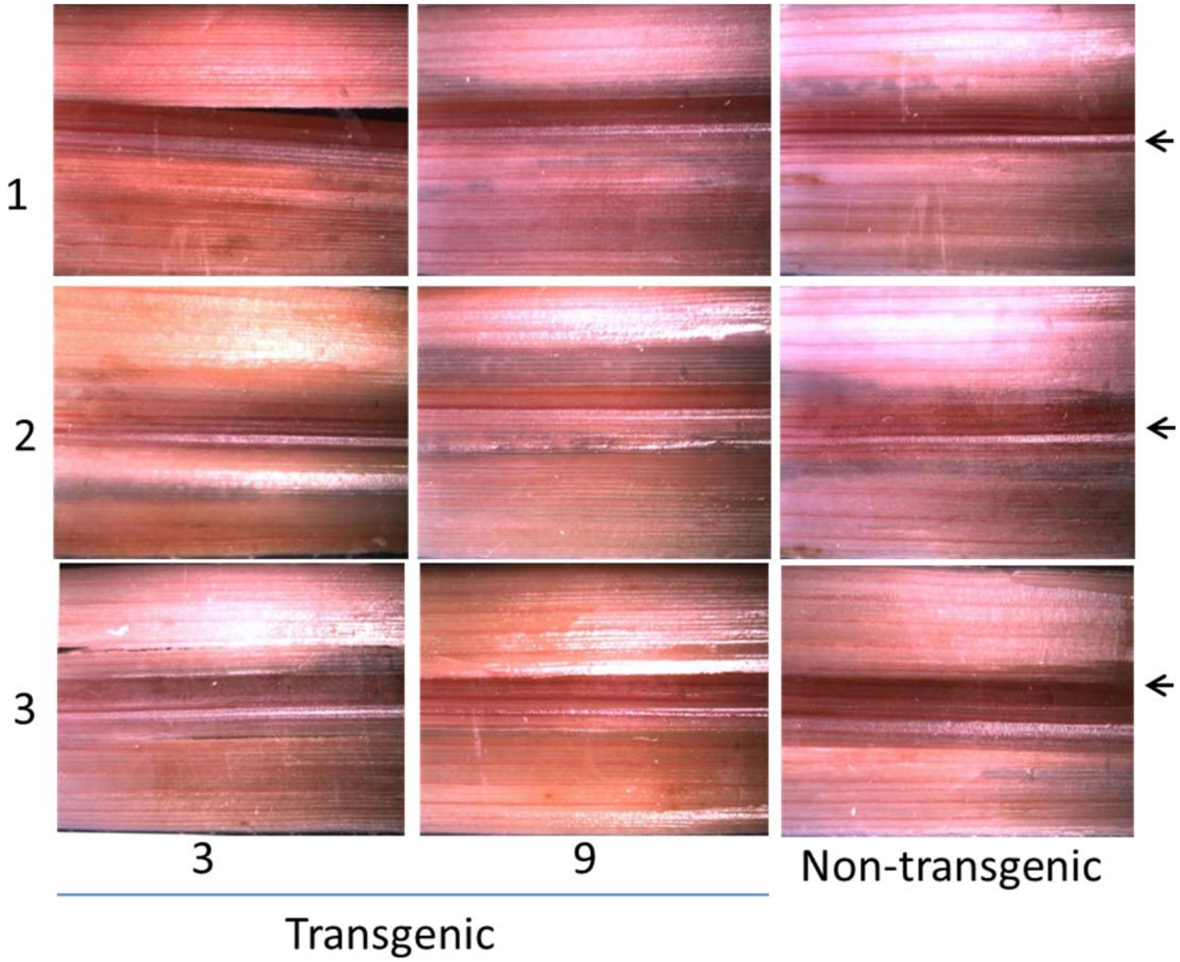

Supplementary Figure 8 Histochemical detection of lignin in leaves of *PvERF001* overexpressing lines compared to non-transgenic control. The phloroglucinol-HCl staining was done on leaves from three independent tillers and the pictures were taken at 2x magnification.

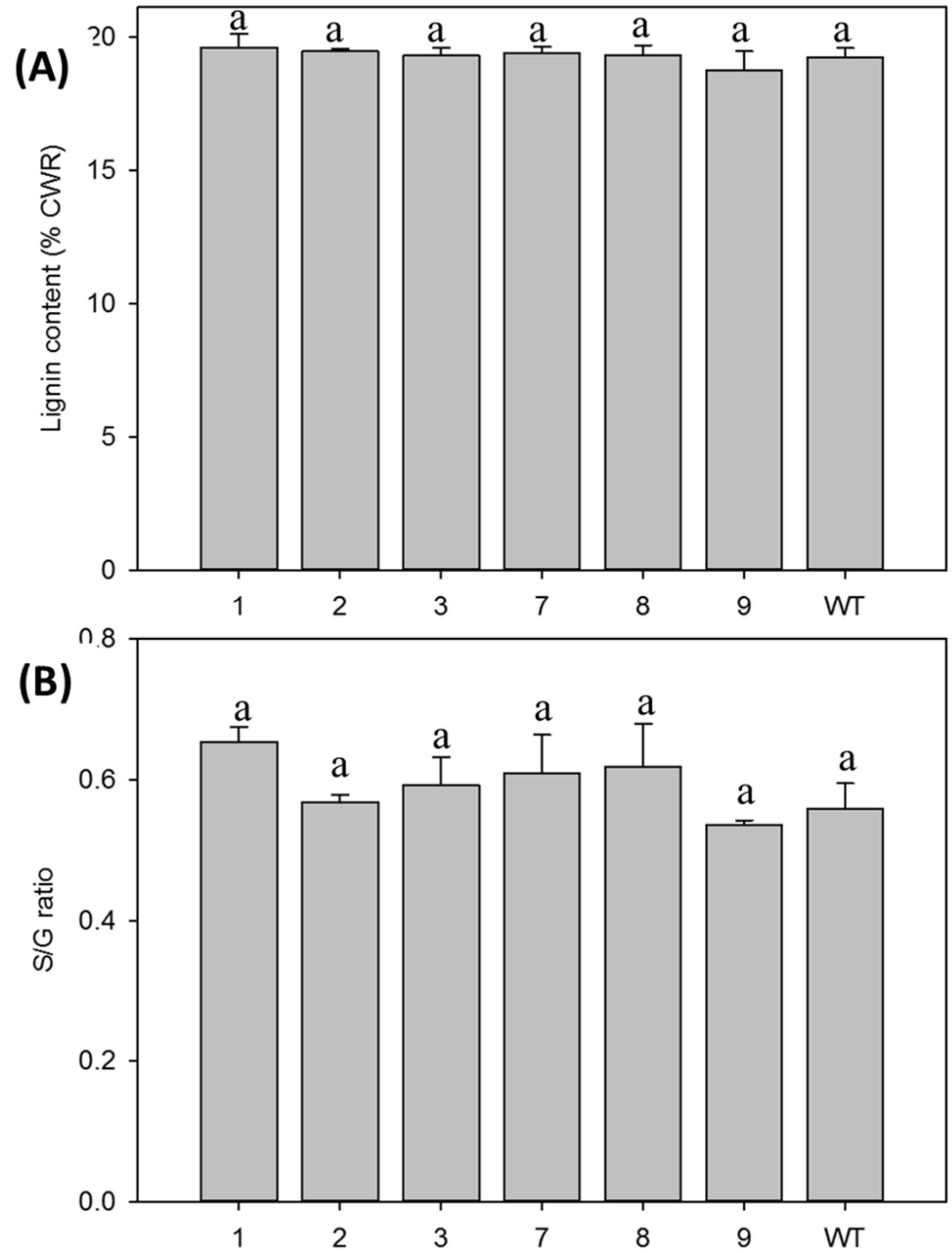

Supplementary Figure 9 Lignin content (A) and S/G ratio (B) of transgenic and non-transgenic (WT) switchgrass lines as determined via py-MBMS. Bars represent the average of the replicates ± standard error. CWR, cell wall residues. Bars represented by different letters are significantly different at $P \leq 0.05$ as tested by LSD method with SAS software (SAS Institute Inc.).
